# Curvulin and Phaeosphaeride A from *Paraphoma* sp. VIZR 1.46 Isolated from *Cirsium arvense* as Potential Herbicides

**DOI:** 10.3390/molecules23112795

**Published:** 2018-10-28

**Authors:** Ekaterina Poluektova, Yuriy Tokarev, Sofia Sokornova, Leonid Chisty, Antonio Evidente, Alexander Berestetskiy

**Affiliations:** 1 All-Russian Institute of Plant Protection, Podbelskogo 3, 196608 Pushkin, Russia; e.poluektova@vizr.spb.ru (E.P.); ytokarev@vizr.spb.ru (Y.T.); svsokornova@vizr.spb.ru (S.S.); 2 Research Institute of Hygiene, Occupational Pathology and Human Ecology, Federal Medical Biological Agency, p/o Kuz’molovsky, 188663 Saint-Petersburg, Russia; mehrn.q2@gmail.com; 3 Department of Chemical Sciences, University of Naples Federico II, Complesso Universitario Monte S. Angelo, Via Cintia 4, 80126 Napoli, Italy; evidente@unina.it

**Keywords:** *Paraphoma* sp. VIZR 1.46, curvulin, phaeosphaeride A, phytotoxins, herbicidal potential

## Abstract

*Phoma*-like fungi are known as producers of diverse spectrum of secondary metabolites, including phytotoxins. Our bioassays had shown that extracts of *Paraphoma* sp. VIZR 1.46, a pathogen of *Cirsium arvense*, are phytotoxic. In this study, two phytotoxically active metabolites were isolated from *Paraphoma* sp. VIZR 1.46 liquid and solid cultures and identified as curvulin and phaeosphaeride A, respectively. The latter is reported also for the first time as a fungal phytotoxic product with potential herbicidal activity. Both metabolites were assayed for phytotoxic, antimicrobial and zootoxic activities. Curvulin and phaeosphaeride A were tested on weedy and agrarian plants, fungi, Gram-positive and Gram-negative bacteria, and on paramecia. Curvulin was shown to be weakly phytotoxic, while phaeosphaeride A caused severe necrotic lesions on all the tested plants. To evaluate phaeosphaeride A’s herbicidal efficacy, the phytotoxic activity of this compound in combination with five different adjuvants was studied. Hasten at 0.1% (*v*/*v*) was found to be the most potent and compatible adjuvant, and its combination with 0.5% (*v*/*v*) semi-purified extract of *Paraphoma* sp. VIZR 1.46 solid culture exhibited maximum damage to *C. arvense* plants. These findings may offer significant importance for further investigation of herbicidal potential of phaeosphaeride A and possibly in devising new herbicide of natural origin.

## 1. Introduction 

The search of new biologically active compounds and investigation of their mode of action is an important task for the development of new pesticides, including herbicides [1,2]. Natural products are the main sources of the molecules with new structures and original targets of action [3]. Natural phytotoxic metabolites can serve as lead compounds or as templates for the development of novel synthetic herbicidal agents and for mode of action studies [4,5]. Many have been isolated from fungal plant pathogens [6,7,8]. *Phoma*-like fungi include numerous plant pathogenic species and some of them were found to produce phytotoxic secondary metabolites. For instance, herbarumin I, a phytotoxin of *Phoma herbarum*, was reported as a highly effective inhibitor of the radicle growth of *Amaranthus hypochondriacus* seedlings [9]. Phytotoxins of *Phoma macrostoma*, macrocidin A and macrocidin B were found to be active ingredients of the extracts obtained from liquid culture of this fungus which are used as a bioherbicide for Canada thistle [10]. *Phoma exigua* var. *exigua* was proposed as a mycoherbicide for the biocontrol of the perennial weeds *Sonchus arvensis* and *Cirsium arvense*. When grown in liquid and solid cultures the fungus produced *p*-hydroxybenzaldehyde, cytochalasins B, F, Z2 and Z3, and deoxaphomin with potential herbicidal activity [11]. Two phytotoxic tetrasubstituted furopyrans, named chenopodolans A and B [12] and a phytotoxic unrearranged *ent*-pimaradiene, named chenopodolin [13], were isolated from culture filtrate of *Phoma chenopodiicola*, a pathogen of the annual weed *Chenopodium album*.

Besides the appropriate activity against the target species, natural herbicides are desired to be safe and selective for their direct use in crop protection [4,5,14,15,16]. Therefore, the wide range of bioassays should be used additionally to phytotoxicity testing of natural compounds. Moreover, the most of natural phytotoxins cannot overcome the cuticle barrier of the plant leaves and in many cases leaf wounding or infiltration are used to obtain a phytotoxic effect in currently used bioassays [17]. Efficient delivery of the active ingredient to the target sites of the plant is required to achieve phytotoxic effect. 

A suitable adjuvant could increase the herbicide uptake by influencing leaf wettability, droplet contact area and penetration, following from reduced surface tension and contact angle of droplets on leaf surfaces [17,18]. Nonionic and anionic surface-active agents and various oils are the most used adjuvants [18,19]. Many research works have focused on using adjuvants to improve the efficacy of herbicide agents, so that their rates could be reduced [19]. Some solvents, for instance dimethylsulfoxide (DMSO), can also increase permeability of lipophilic molecules through the leaf cuticle [20]. Currently, there is no data available on the use of adjuvants together with natural phytotoxins to improve their activity on intact leaves of weeds.

*Paraphoma* sp. VIZR 1.46 strain (earlier reported as *Phoma* sp. No. 19) was isolated from leaves of Canada thistle (*C. arvense*). The extracts from solid and liquid culture of this fungus were shown to be phytotoxic and had different metabolic profiles [21]. In this paper, the production, isolation and biological characterization of phytotoxic metabolites produced by this fungus are described. Additionally, the effect of five commercial adjuvants was studied in order to evaluate the herbicidal potential of one of the main phytotoxins produced by *Paraphoma* sp. VIZR 1.46.

## 2. Results and Discussion

### 2.1. Fungal Identification

The fungus was isolated from diseased leaves of C. arvense. In vitro, the fungus did not sporulate on a range of agar media so molecular tools were used for its identification. Analysis of the nucleotide sequences of internal transcribed spacer (ITS), large subunit (LSU) and translation elongation factor (TEF) regions of DNA suggested that strain VIZR 1.46 is phylogenetically close to *Phoma*-like fungi, particularly, *Paraphoma chrysanthemicola*, *Paraphoma radicina* and *Paraphoma vinacea*. The phylogenetic tree of LSU was used for species of closely related genera to assess the placement of the isolates within the family *Phaeosphaeriaceae* [22] (see Supplementary Materials, Figures S1 and S2). 

Based on a basic local alignment search tool (BLAST) search of National Center for Biotechnology Informations (NCBIs) GenBank nucleotide database sequences of *Paraphoma* sp. VIZR 1.46 were identical to *P. chrysanthemicola* CBS 522.66, *P. radicina* CBS 102,875 and *P. vinacea* UMPV 003. However, analysis of LSU region of these strains indicated that *P. chrysanthemicola* CBS 522.66, *P. radicina* CBS 102,875 and *P. vinacea* UMPV 003 were identical, while the sequence of our strain differed from them in four point mutations (see Supplementary Materials, Figure S3).

On oatmeal agar (OA) colony diameter reached 57 mm after two weeks; its pigmentation was olivaceous gray at the margin and dark red in the center while aerial mycelium was white grayish. On malt extract agar (MA) colony diameter was about 44 mm after two weeks; the pigmentation was dark gray at the margin and olivaceous gray in the center; aerial mycelium white grayish. Colony morphology was very similar to *Paraphoma vinacea* [23] and to *Phoma sanguinolenta* [24], both known as pathogens of some *Asteraceae* plants.

Based on the molecular and morphological data the isolate VIZR 1.46 was placed to the genus *Paraphoma.* It was clearly shown that *Paraphoma* sp. VIZR 1.46 differs from the most common *Phoma*-like fungi hosted by *C. arvense: Didymella cirsii* (*Phoma cirsii*), *Pleospora herbarum* (*Didymella herbarum*), *Boeremia hedericola* (*Phoma hedericola*), *Didymella macrostoma* (*Phoma macrostoma*), *Boeremia exigua* and *Plenodomus libanotidis* (*Phoma sanguinolenta; Leptosphaeria rubella*), *Stagonospora cirsii* and *Phyllosticta cirsii* [25,26]. Some of these fungi are known as producers of phytotoxic metabolites: macrocidins [10], phyllostictines [26] and stagonolides [27]. Analysis of the ITS sequence of *Paraphoma* sp. VIZR 1.46 suggests that it significantly differs from *Phyllosticta cirsii* [28].

### 2.2. Purification and Identification of Phytotoxins

**Figure 1:**
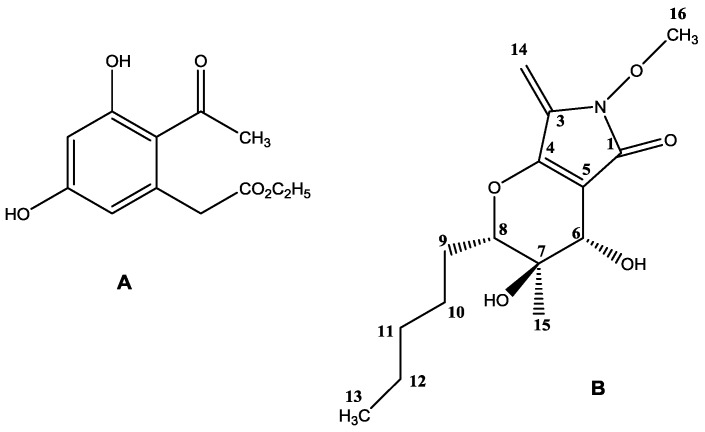
Structures of curvulin (**A**) and phaeosphaeride A (**B**).

Fractionation of *Paraphoma* sp. VIZR 1.46 liquid culture extract (65 mg/L) by column chromatography (CC) and preparative thin layer chromatography (PTLC) gave the main phytotoxic metabolite (1 mg/L) as described in details in the Materials and Methods section. Curvulin (ethyl 2-(2-acetyl-3,5-dihydroxyphenyl)acetate) (Figure 1A) was identified by electrospray ionization mass spectrometry (ESI-MS), nuclear magnetic resonance (NMR) and ultraviolet (UV) spectroscopic methods (see Supplementary Materials, Figures S4–S7) with those (mass spectra (MS) and ^1^H- and ^13^C-NMR) reported in its isolation, together with *O*-methylcurvulinic acid, from *Drechslera indica* [29,30]. This fungus was proposed as a mycoherbicide for *Portulaca oleracea*, a spreading weed causing serious losses for important agrarian crops, such as cotton, corn, rice and potatoes. *D. indica* was also pathogen for *Amaranthus spinosus*, another noxious weed, and rape, a valuable source of cooking oil [31]. Curvulin had been previously isolated from *Curvularia siddiqui* and its structure in that time was characterized by chemical methods and infrared (IR) and UV spectra [30,31]. Curvularin was also isolated from *Curvularia lunata* together with methyl 2-acetyl-3,5-dihydroxyphenylacetate, methyl 2-acetyl-5-hydroxy-3-methoxyphenylacetate and 4-epiradicinol [32]. The identification of curvulin was also supported from the data from its ESI MS spectrum (Figure S5) which, beside the expected sodium cluster [M + Na]^+^ and the pseudomolecular ion [M + H]^+^ at *m*/*z* 261 and 239, respectively, showed the significant fragmentation ions, not previously reported, at *m*/*z* 211, 197, 193, 165 generated from the pseudomolecular ion by alternative loss of C_2_H_4_, CH_2_CO, C_2_H_5_OH, C_2_H_5_CO_2_H, while the ion observed at *m*/*z* 123 was produced by the latter ion at *m*/*z* 165 by loss of CH_2_CO. When curvulin was isolated from both *Curvularia siddiqui* and *Curvularia lunata* no any biological activity was reported but when it was more recently isolated from two *Bipolaris* strains and two *Eichhornia macrophytes* species, together with spirostaphylotrichin R and U, the three metabolites showed antileishmanial activity [33]. However, this is the first report on the isolation of curvulin from a *Paraphoma* sp. 

The extract obtained from the solid culture of the fungus was fractionated by chromagraphic methods as described in the Materials and Methods section. It resulted in purification of another phytotoxic metabolite (130 mg/kg). ^1^H- and ^13^C-NMR data as well as mass spectrum of this compound are very similar to the data reported for phaeosphaeride A (3,4-dihydroxy-6-methoxy-3-methyl-7-methylene-2-pentyl-3,4,6,7-tetrahydropyrano[2–*c*]pyrrol-5(2*H*)-one) (Figure 1B), isolated from the endophyte FA 39 closely related to *Phaeosphaeria avenaria* as an inhibitor of STAT3-dependent signaling [34]. An extensive spectroscopic investigation was carried out in the present work, on the phaeosphaeride A isolated from *Paraphoma* sp. VIZR 1.46 and its ^1^H, hydrogen correlated spectroscopy (^1^H-COSY), ^1^H, carbon heteronuclear multiple-quantum correlation (^13^C-HMQC), hydrogen and carbon heteronuclear multiple bond correlation (^1^H-, ^13^C-HMBC) and ^1^H, hydrogen rotating-frame Overhauser spectroscopy (^1^H-ROESY) NMR spectra [35] (Supplementary Materials, Figures S13–S17) were carefully investigated. In particular, the ^1^H-, ^13^C-HMBC (Figure S15) showed that H-6 correlates to C-4, C-5 and C-8 and this confirmed the bicyclic structure of phaeosphaeride A, OH at C-7 correlates to CH_3_-15, C-8 and OH at C-6 correlates to C-7. ^1^H, ^1^H-COSY (Figure S13) data showed the same correlations for three spin systems as observed in phaeosphaeride A as reported by Maloney and colleagues [34] while ^1^H-, ^1^H-ROESY spectrum (Figures S16 and S17b) of phaeosphaeride A showed correlations between CH_2_-14 (δ 4.98) and CH_3_-16 (δ 3.80), H-6 (δ 3.87) and H-8 (δ 4.08) as observed and above reported in the nuclear Overhauser effect (NOE) ^1^H-NMR spectrum of in phaeosphaeride A [34] but differed as no correlation was noted between OH-7 (δ 4.92) and OH-6 (δ 5.42).

The structure originally assigned to phaeosphaeride A from Maloney and co-workers was successively corrected as a result of the first synthetic attempts [36,37] and by its total enantioselective synthesis also carried out to investigate in more depth its biological activity and perform structure-activity relationships studies [38,39]. These results, which also allowed assigning its absolute configuration as reported in Figure 1B, were also supported from the crystallographic analysis carried out on the natural metabolites isolated from *Phoma* sp. (then reclassified as *Paraphoma* sp.) [40]. All the extensive synthetic and crystallographic work as well as the further biological investigation carried out on phaeosphaeride A and natural and synthetic analogues were recently reviewed [41].

The structure of phaeosphaeride A isolated from *Paraphoma* sp. VIZR 1.46 assigned to metabolite reported on the Figure 1B [41] was also supported by the data observed in its ESI MS spectrum (Figure S9), which showed, the expected sodium cluster [M + Na]^+^, the pseudomolecular ion [M + H]^+^ and the significant fragmentation ions, which are generated by this latter by successive losses of two water molecules [M + H-H_2_O]^+^ and molecules [M + H-2H_2_O]^+^, at *m*/*z* 320, 298, 280 and 262, respectively.

There are other known phytotoxic spirocyclic γ-lactames, e.g., spirostaphylotrichines isolated from phytopathogenic fungi infecting wheat and some weeds from *Poaceae* [42]. Curvupallides are another group of structurally related metabolites produced by phytopathogenic fungus *Curvularia pallescens* [43]. Paraphaeosphaerides A–C isolated from *Paraphaeosphaeria neglecta* FT462 exhibit another group of structurally related compounds with different bioactive properties [44]. However, this is the first report on phaeosphaeride A as a potent fungal phytotoxin.

### 2.3. Biological Activity

**Figure 2:**
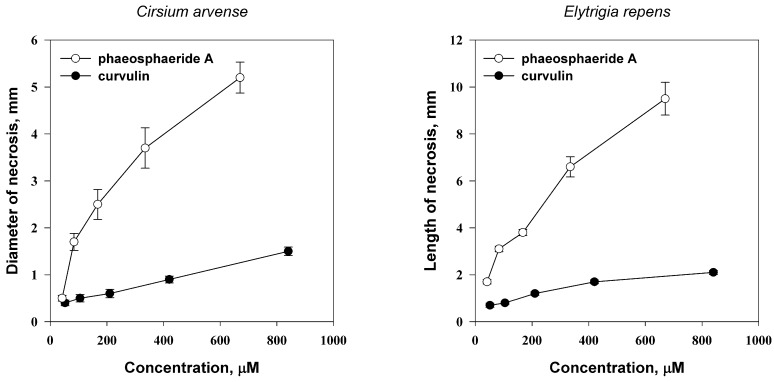
Phytotoxic activity of curvulin phaeosphaeride A for *Cirsium arvense* (**left**) and *Elytrigia repens* (**right**) in different concentrations. Bars indicate standard deviation at *p* = 0.05.

Phytotoxic activity of curvulin and phaeosphaeride A was assayed on wounded leaf segments of *C. arvense* and *Elytrigia repens.* Both toxins caused necrotic lesions on leaves of the tested weeds within 24 h post treatment. The concentration of phaeosphaeride A required for induction of considerable leaf necrotic lesions (necrosis ≥ 2 mm in diameter) of 67 μM and 84 μM for *C. arvense* and *E. repens*, respectively. Curvulin showed significant phytotoxic activity at higher concentrations of ≥840 μM (Figure 2). Weak phytotoxic activity of curvulin against *A. spinosus* and *P. oleraceae* was reported earlier [33].

**Figure 3:**
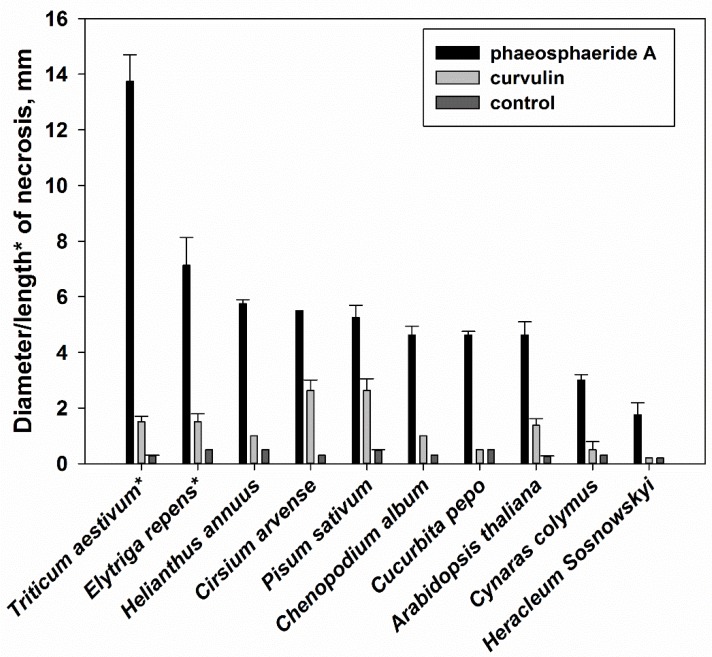
Phytotoxic selectivity of curvulin and phaeosphaeride A. tested at 840 μM and 335 μM, respectively (10 μL/leaf disc/leaf segment). Bars indicate standard deviation at *p* = 0.05. The length of necrosis was measured when leaf segments of *T. aestivum* and *E. repens* were used, while for other plant species the diameter of the necrosis was estimated.

Phytotoxic activity of phaeosphaeride A at 335 μM concentration was not selective. Surprisingly, *E. repens* and *Triticum aestivum* (both from *Poaceae* family) were more sensitive to phaeosphaeride A than the host plant of the producing fungus, *C. arvense* (Figure 3). *C. arvense* and *Pisum sativum* were the most sensitive to curvulin at the concentration 840 μM while other plant species were less susceptible to this toxin (Figure 3).

**Figure 4:**
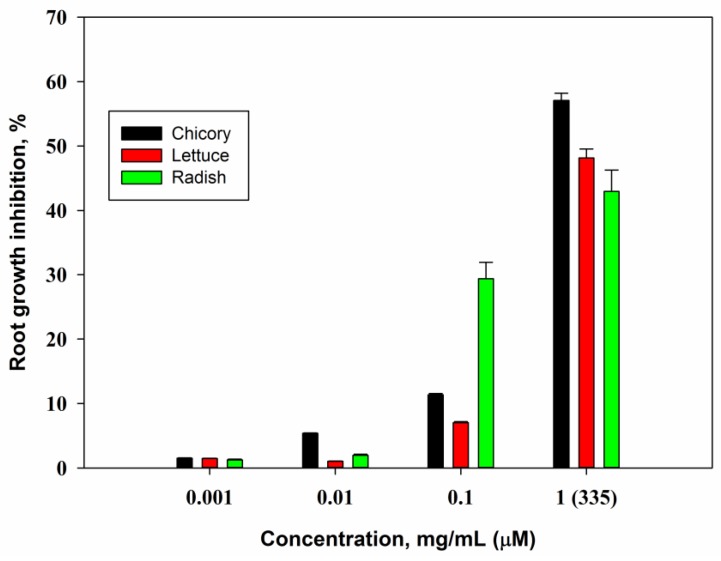
Phytotoxic activity of phaeosphaeride A in the root inhibition bioassay. Bars indicate standard deviation at *p* = 0.05.

At the maximal concentration of 1 mg/mL (335 μM) phaeosphaeride A inhibited root growth of seedling of lettuce, chicory and radish at the level about 50% compared with control. At the concentration of 0.1 mg/mL the root growth of radish only was inhibited considerably (Figure 4). 

Curvulin was nontoxic in this bioassay at all the concentrations tested. It is known that many phytotoxins inhibit root growth, e.g., herbarumin I from *P. herbarum* and stagonolide A from *S. cirsii* demonstrated half maximal inhibitory concentration (IC_50_) at the concentrations 50 μM and 5 μM respectively [9,45]. Comparing to these compounds the phytotoxic activity of phaeosphaeride A is weak. This level of phytotoxic activity of phaeosphaeride A is comparable to another tetramic acide derivative, spirostaphylotrichin A [42]. 

None of the two tested phytotoxins showed antimicrobial activity when assayed on the eight bacteria and two fungi at the concentration up to 100 μg/disc. Curvulin was reported to lack antimicrobial activity against *Escherichia coli*, *Staphylococcus aureus*, *Salmonella choleraesuis* and *Bacillus subtilis* [29,33]. It was shown earlier that phaeosphaeride A (identified as phyllostictine B) has no antimicrobial and antifungal activity when tested on *Geotrichum candidum*, *Lactobacillus* sp. and *E. coli* [26].

Phaeosphaeride A was shown to be weakly toxic to *Paramecia caudatum*. At the relatively high concentration of 40 μM 40% of ciliates stopped movement 3 h post treatment. These data were in accordance to negligible activity of this compound on brine shrimp (*Artemia salina* L.) larvae [26]. Curvulin was completely inactive to *Paramecium caudatum*.

### 2.4. Effects of Leaf Wounding, Solvent and Adjuvants on Phytotoxic Activity of Phaeosphaeride A

**Figure 5:**
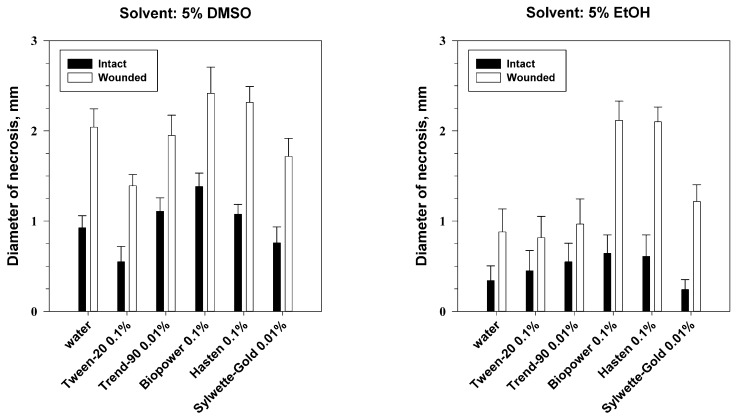
Effect of leaf damage, solvent and adjuvants on phytotoxicity of phaeosphaeride A on leaf discs of *C. arvense*. Least significant difference (LSD) _0.05_ = 0.53, bars indicate standard deviation at *p* = 0.05.

Analysis of variance showed significant effects of adjuvants (*p* < 0.001) as well as wounding (*p* < 0.001) on phytotoxic activity of phaeosphaeride A on leaf discs of *C. arvense*. The interaction of these factors was insignificant. Wounding the leaf discs of *C. arvense* gave at least 2-fold increase of phytotoxic activity of phaeosphaeride A comparing to its effect on the intact leaf discs (Figure 5). The effect of the solvent was pronounced only with a combination with the factor of leaf wounding (*p* < 0.001) or adjuvant (*p* < 0.01) (Table S2).

When the toxin was dissolved in ethanol (EtOH) its phytotoxic activity in the combination with Hasten or Biopower was significantly higher than the activity of phaeosphaeride A alone. Combinations of phaeosphaeride A, which was dissolved in DMSO, with Biopower and Hasten gave insignificant increase of its activity on both wounded and intact leaf discs of *C. arvense*. On the other hand, the addition of Tween-20 and Sylwett-Gold to the toxin solution slightly decreased phytotoxicity of phaeosphaeride A for *C. arvense* (Figure 5).

This experiment demonstrated that certain adjuvants can definitely inhibit (Tween-20) or to promote (Biopower and Hasten) the phytotoxic activity of phaeosphaeride A on leaf discs of *C. arvense*. The selection of adjuvants is species-specific: they are able to promote and inhibit the uptake of herbicidal compounds in certain plants [46]. According to the manufacturer, Hasten is a blend of esterified vegetable oil and non-ionic surfactants, however, little is known on efficacy of this adjuvant. A number of adjuvants enhanced the control of *C. arvense* by chemical herbicides, and there is some evidence that oil-based adjuvants are especially useful. Petroleum oil adjuvant added to clopyralid at 0.14 kg/ha increased Canada thistle control to that achieved by clopyralid at 0.21 kg/ha plus desmedipham/phenmedipham plus endothall [47]. Methylated seed oil was the best adjuvant to tribenuron to enhance its efficacy against common cocklebur and Canada thistle [48]. To our knowledge, there is a few of studies on effects of adjuvants on herbicidal activity of natural compounds. For instance, herbicidal activity of acetic acid, in particular, against *C. arvense* was improved by canola oil (0.25% *v*/*v*) as an adjuvant [49]. Biopower (sodium lauryl sulfate) was shown to be useful in a number of herbicidal compositions for control of various weeds [50,51].

### 2.5. Effect of Adjuvants on Herbicidal Activity of Phaeosphaeride A

**Figure 6:**
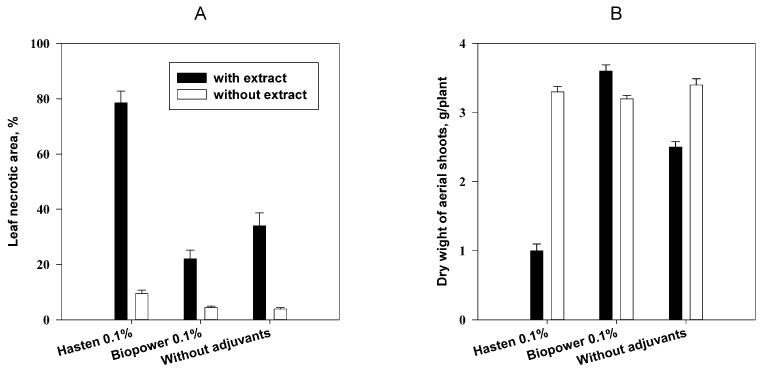
Effect of adjuvants on herbicidal activity of 0.5% semi-purified extract from solid culture of *Paraphoma* sp. VIZR 1.46 on *C. arvense* plants 48 h after treatment: (**A**) level of leaf damage (LSD _0.05_ = 11.0), (**B**) dry biomass of aerial shots (LSD _0.05_ = 1.8), bars indicate standard deviation at *p* = 0.05.

Hasten and Biopower were evaluated with a combination of 0.5% semi-purified extract of the fungal solid culture containing about 50% of phaeosphaeride A. This semi-purified extract without adjuvants was shown to have weak herbicidal activity on aerial shoots of *Cirsium arvense* damaging about 35% of leaf surface. This effect was accompanied with insignificant decrease of dry weight of aerial shoots. Herbicidal effect of the extract with Biopower was considerably lower. In contrast, treatment of the plants with the extract with Hasten resulted in damage of ca. 80% of leaf surface and 3-fold loss of dry weight comparing to control plants (Figure 6).

This experiment supported Hasten as the adjuvant compatible with phaeosphaeride A. In contrast to the experiment with leaf discs of *C. arvense*, the adverse effect of Biopower on phytotoxic activity of phaeosphaeride A on the whole plants was observed. It can be explained by different application techniques used in this and previous experiments affected droplet size and penetration of the toxin in the leaf tissues. Moreover, both adjuvants were applied at the non-phytotoxic concentration (0.1% *v*/*v*) that is lower than recommended concentrations of 0.5% and 0.25% for Hasten and Biopower respectively [52,53]. In the future, manipulation with the phytotoxin formulation including increasing concentration of adjuvants and addition of humectants like glycerol could enhance phytotoxicity and efficacy of phaeosphaeride A as a natural herbicidal compound [46].

## 3. Material and Methods

### 3.1. General Experimental Procedures

Optical rotations were measured using AA-55 polarimeter (Optical Activity, Ramsey, United Kingdom) in CH_2_Cl_2_. The UV spectra were measured in MeCN on a DU800 UV-vis spectrophotometer (Beckman Coulter, Brea, CA, USA). ^1^H- and ^13^C-NMR spectra were recorded at 400 and 100 MHz, respectively, on a WM-400 spectrometer (Bruker, Billerica, MA, USA) in CDCl_3_, unless otherwise noted. The same solvent was used as the internal standard. COSY, ROESY, DEPT, HMBC and HMQC experiments [35] were recorded using Bruker microprograms. ESI-MS were obtained on a TSQ Quantum Access™ mass spectrometer (Thermo Fisher Scientific, Waltham, MA, USA). Analytical and PTLC were performed on silica gel plates (Kieselgel 60, F254, Merck, Darmstadt, Germany, 0.25 and 0.5 mm, respectively). The spots were visualized exposure to UV radiation (254 nm). Column chromatography was performed on a silica gel (Kieselgel 60, 0.063−0.200 mm, Merck) Reverse phase column chromatography was carried out using a C-18ec Chromabond 10-g cartridge (Macherey-Nagel, Düren, Germany). Medium pressure liquid chromatography (MPLC) was performed with a Sepacore system (Büchi, Flawil, Switzerland) equipped with a UV-detector. Fractionation was performed with a PuriFlash SiHC cartridge (SiOH, 40 g, Interchim, Montluçon, France).

### 3.2. Fungal Strain

The fungus was isolated from necrotic leaf lesions in naturally infected *C. arvense*. The herbarium samples were collected from the Khabarovskiy region (Russian Federation) in 2006. The culture of the fungus *Paraphoma* sp. VIZR 1.46 was stored on potato-glucose-agar (PGA) slants in test tubes at 4 °C. For the initial culture production, small pieces of mycelium were aseptically transferred to PGA plates and incubated for 14 days at 24 °C in the dark. Mycelial plugs (about 5 mm in diameter) from the colony margins were used for inoculation of liquid and solid media. The DNA of the fungus, was extracted using Sambrook method [54] with Ultraclean Microbial DNA isolation kit (Mo Bio Laboratories, Carlsbad, CA, USA). For nucleotide sequence comparisons fragments of three loci were analysed: LSU, ITS, and TEF. Amplification of LSU was conducted utilizing the primer combination LR0R [55] and LR5 [56], EF1-728f and EF1-986R were used for TEF sequencing and the primer pair ITS1 and ITS4 [57] for ITS region. The PCRs were performed in a Tercik Thermal Cycler (DNA-Technology, Moscow, Russia) in a total volume of 25 μL. Sequence products were purified and subsequently separated and analysed on an ABI Prism 3500 DNA Sequencer (Applied Biosystems, Foster City, CA, USA). Consensus sequences were computed from the forward and reverse sequences using the BioEdit v. 7.2.5 software package (Ibis Biosciences, Carlsbad, CA, USA). The consensus sequences were deposited in GenBank (GenBank accession numbers KT289378, KT289379, KT289380).

### 3.3. Production and Purification of Phytotoxic Metabolites

#### 3.3.1. Liquid Culture

YMG growth medium contained yeast extract (4 g), malt extract (10 g), glucose (10 g) and water (1 L). For seed culture the medium (250 mL per 1000-mL conical flask) was inoculated with two agar plugs cut off the 2-week fungal colony produced on PGA. The seed liquid cultures were incubated on the orbital shaker at 180 rpm (revolution per minute) for seven days. The fermenter (20 L volume) containing 15 L of the same medium was inoculated with a suspension of the 7-day seed culture. After 10 days of the fermentation at 24 °C the culture fluid was separated from the mycelium by filtering through cheesecloth using a vacuum pump. The culture filtrates were extracted with ethyl acetate (EtOAc) (2 × 5 L). The organic extracts were combined, dehydrated with Na_2_SO_4_, filtered and evaporated under reduced pressure to give a residue 970 mg (yield 65 mg/L). The extract was fractionated by column chromatography on silica gel (Merck 60, 0.040–0.063 mm) eluted with *n*-hexane-EtOAc from 100:0 to 0:100 (*v*/*v*) in order of their increasing polarity, yielding 10 groups of homogenous fractions. The residues of C and D fraction groups showed phytotoxic activity and were fractionated on silica gel PTLC plates (Merck 60, F_254_, 0.50 mm,) with the eluent system *n*-hexane-EtOAc (6:4, *v*/*v*). This gave 15 mg of one major metabolite (R_f_ 0.6, yield 1 mg/L) which was identified, as below reported, as curvulin (Figure 1A).

#### 3.3.2. Solid Culture

Erlenmeyer flasks of 1 L volume (totally 18 flasks) containing 150 g of pearl barley and 100 mL of water were autoclaved at 121 °C for 30 min. Each flask was inoculated with two agar plugs of the fungus. To avoid the balling of the infected substrate, the flasks were stirred up once every 2 days. After 30 days of incubation at 24 °C in the dark the culture was dried with a stream of sterile air for 2 days at a room temperature. The dried material (2.1 kg) was extracted with the mixture acetone–water (1:1 *v*/*v*, 7 L). Acetone was evaporated under reduced pressure at 40 °C. The water residue was defatted with *n*-hexane (3.5 L) and then extracted with EtOAc (3 × 2.5 L). 

The combined organic extracts were dried (Na_2_SO_4_), filtered and evaporated to dryness under reduced pressure at 40 °C, yielding the oily residue (4 g). The latter was fractionated by reverse-phase column chromatography using a cartridge, eluted with 0.1% (*v*/*v*) of formic acid in deionized water followed by 25% MeCN in 0.1% formic acid (*v*/*v*). The final elution was performed with 50% MeCN in 0.1% formic acid. The oily residue (1 g) obtained from this fraction was further purified by MPLC and the cartridge was eluted with n-hexane-EtOAc (gradient 0–80% *v*/*v* EtOAc) at a flow rate of 50 mL/min, collecting fractions of 10 mL. The main metabolite was afforded (R_f_ 0.5 in CHCl_3_–iso-PrOH 9:1 *v*/*v*, 351 mg, 130 mg/kg) and identified, as below reported, as phaeosphaeride A (Figure 1B).

### 3.4. Compound Characterization

*Curvulin* (Figure 1A). White crystalline compound, UV λ_max_ 220, 275, 310 nm; ^1^H-NMR δ 1.26 (t), 2.58 (s), 3.86 (s), 4.19 (q), 6.28 (s), 6.34 (s), 8.17 (s), 12.83 (s); ^13^C-NMR δ 13.9, 31.7, 41.7, 61.2, 103.0, 112.7, 115.7, 137.3, 161.6, 165.6, 170.6, 203.0; ESI-MS, *m*/*z* 261 [M + Na]^+^; 239 [M + H]^+^, 211 [M + H-C_2_H_4_]^+^, 197 [M + H-CH_2_CO]^+^, 193 [M + H-C_2_H_5_OH]^+^, 165 [M + H-C_2_H_5_CO_2_H]^+^, 123 [M + H-CH_2_CO-HCO_2_C_2_H_5_]^+^.

*Phaeosphaeride A* (Figure 1B). Yellowish glass; [α]^25^_D_ −108.33 (c 0.06, CH_2_C_l2_); UV λ_max_ 262 nm, ^1^H- NMR (DMSO-*d*_6_) δ 0.86 (t, *J* = 6.4 Hz, 3H), 1.19 (s, 3H), 1.27 (m, 2H), 1.49 (m, 2H), 1.52 (m, 2H), 1.82 (m, 2H), 3.80 (s, 3H), 3.87 (d, *J* = 12.0 Hz, 1H), 4.09 (d, *J* = 12.0 Hz, 1H), 4.91 (s, 1H), 4.98 (s, 2H), 5.43 (d, *J* = 12.0 Hz, 1H); ^13^C-NMR (DMSO-*d*_6_) δ 13.9, 20.4, 20.0, 26.1, 27.6, 30.9, 63.8, 64.4, 71.0, 86.3, 90.8, 104.8, 137.1, 155.3, 166.5; ESI-MS, *m*/*z* 320 [M + Na]^+^, 298 [M + H]+, 280 [M + H-H_2_O]^+^, 262 [M + H-2H_2_O]^+^.

### 3.5. Biological Assays

Phytotoxic activity was studied using leaf disc-puncture bioassay as previously described [10]. The purified metabolites were tested at a range of concentrations from 0.125 to 2 mg/mL (42–840 μM). The samples were dissolved in EtOH and adjusted to the desired concentration with distilled water. The final concentration of the solvent was 5% *v*/*v*, which was not phytotoxic. The leaf discs (1 cm diameter) of dicotyledonous plants and the leaf segments of monocotyledonous plants (2 cm length) were collected from well expanded leaves, placed on the moistened general purpose filter paper (“F”, Bashkhimservis, Russia) in transparent plastic boxes and wounded in the center with a sharp needle. The selectivity of compounds curvulin (at concentration 840 μM) and phaeosphaeride A (at concentration 335 μM) was studied using plants of different families: *Cirsium arvense*, *Helianthus annuus*, *Cynara scolymus* (*Asteraceae*), *Arabidopsis thaliana (Brassicaceae*), *Heracléum sosnówskyi* (*Umbellíferae*), *Pisum sativum (Fabaceae*), *Chenopodium album* (*Chenopodiaceae*), *Cucurbita pepo (Cucurbitaceae*), *Elytrigia repens*, *Triticum aestivum* (*Poaceae*). These concentrations were chosen based on preliminary phytotoxicity tests. Droplets (10 μL) of the test solution were applied on the discs and then incubated for two days under artificial light (12 h day/12 h night) and at a controlled temperature (24 °C day/20 °C night). Due to different leaf shape the diameter and length of necrotic lesions was measured when used leaf discs of dicot species and leaf segments of monocot plant species respectively. Ten replicate leaf discs/segments were used for each treatment. Experiments were conducted twice. 

For root growth inhibition bioassay seeds of radish (*Raphanus sativus*), lettuce (*Lactuca sativa*), chicory (*Cichorium endivia*) were surface sterilized with 70% EtOH and incubated on moistened filter paper in a wetness chamber for germination at the temperature 25 °C in the darkness. Seedling with rootlets of 1–2 mm length were soaked in the solutions of phytotoxins (0.001–1 mg/mL), which were prepared as described above, for 1 h, than washed by water and incubated at 25 °C in the darkness. The length of rootlets measured 48 h post treatment. Phytotoxic activity (A) calculated by the formula: $A=100-\left(\frac{\mathrm{LE}48-\mathrm{LEo}}{\mathrm{LC}48-\mathrm{LCo}\;}\;\times 100\right)$, where LE_0_ and LE_48_—means of rootlet length zero and 48 h post treatment in the experiment, LC_0_ and LC_48_—means of rootlet length zero and 48 h post treatment.

The antimicrobial activity of curvulin and phaeosphaeride A was tested using the paper disc technique according to the already described protocol [58]. A preliminary study was conducted with three test microorganisms: Gram-positive (*B. subtilis*) and Gram-negative (*Xanthomonas campestris*) bacteria, and the yeast fungus (*Candida tropicalis*). Additional test organisms were used for characterization of phaeosphaeride A: Gram-positive (*Clavibacter michicagenis*) and Gram-negative bacteria (*Pseudomonas corrugate*, *Erwinia carotovora*, *Pseudomonas syringae* pv. *maculicola*, *P. syringae* pv. *tomato*) mycelial phytopathogenic fungi (*Sclerotinia sclerotiorum* and *Botrytis cinerea*). The studied bacteria were grown on medium, consisted of pancreatic sprat hydrolysate 17.9 g/L, microbiological agar 11.2 g/L, and sodium chloride 7.7 g/L (Microgen, Moscow, Russia), while the fungi were grown on PGA. Up to 100 μg of each metabolite was applied per disc. The fungal and bacterial cultures were incubated at 24 °C and 30 °C, respectively, for 48 h and inhibition zones were measured daily.

*Zootoxic activity assay.* The zootoxic activity was evaluated on *Paramecium caudatum* by using the protocol already described [45]. Both phytotoxins were tested at concentrations of 4, 40, and 400 μM in 5% EtOH, with three replications for each concentration of every compound. The assay was performed in a 24-well plate. A suspension of *P. caudatum* cells (900 μL) and 100 μL of stock solutions of curvulin and phaeosphaeride A prepared in 50% EtOH were poured to each well. In the control treatment, 100 μL of 50% EtOH was added to 900 μL of suspension with paramecia. The culture of paramecia was incubated at 24 ± 1 °C. Toxic effects of curvulin and phaeosphaeride A on paramecia movement was observed after 3 and 30 min, 3 h and 24 h post treatment. The toxicity was expressed as percentage of dead paramecia in reference to the total amount.

### 3.6. Effects of Leaf Wounding, Solvents and Adjuvants on Phytotoxic Activity of Phaeosphaeride A

Four different non-ionic adjuvants Trend-90 (isodecyl alcohol ethoxylate, Du Pont, Geneva, Switzerland), Tween-20 (polyoxyethylene sorbitol ester, Croda Crop., Snaith, United Kingdom), Sylwette-Gold (trisiloksanakoksilate, Chemtura, Philadelphia, PA, USA) and Hasten (ethyl and methyl esters of vegetable oil, Victorian Chemicals, Coolaroo, Australia) and one anionic adjuvant Biopower (sodium lauryl sulphate, Bayer CropScience Limited, Cambridge, United Kingdom) were selected for this experiment. Solutions of the adjuvants were prepared in distilled water in following concentrations (*v*/*v*): 0.1% Tween-20, 0.1% Biopower, 0.01% Trend-90, 0.01% Sylwette-Gold, 0.1% Hasten. Preliminary experiments indicated these concentrations of adjuvants to be nontoxic when tested on leaf discs of *C. arvense* and leaf segments of *E. repens*. The purified compund (0.2 mg) was dissolved in 10 μL of EtOH or DMSO and adjusted to the volume of 200 μL with one of the adjuvant solution or water. A 10 μL droplet of the test solution was applied on each wounded and intact leaf disc/segment of *C. arvense*/*E. repens*. Two days post treatment the diameter/length of necrotic lesions was measured. Ten replicate leaf discs/segments were used for each treatment. 

The herbicidal effect of three formulations of phaeosphaeride A was evaluated on plants of *C. arvense* at the stage of rosette. The underground shoots of the weed (cuttings 5 cm length) were planted in pots with a soil mixture and incubated in a greenhouse at approximately 24 °C for 4 weeks. A semi-purified extract containing approximately 50% of the phytotoxin (determined by HPLC) was obtained by passing the crude extract from solid culture of *Paraphoma* sp. VIZR 1.46 through C18 cartridge as described above. The dry residue (240 mg) of the semi-purified extract was dissolved in 2.4 mL of DMSO. This solution was divided in three parts of 0.8 mL, each of them was diluted to the volume of 16 mL with water or 0.1% solution (*v*/*v*) of the adjuvant (Biopower or Hasten). The final concentrations of the extract and DMSO were 0.5% (*w*/*v*) and 5% (*v*/*v*) respectively. The formulations of phaeosphaeride A were sprayed onto plants with a hand atomizer (6 mL per pot with 3 plants, 3 replicate pots per treatment). The dosage of phaeosphaeride A was about 0.05 mol/m^2^. The 5% DMSO water solution without and with the adjuvants (Biopower or Hasten, 0.1% *v*/*v*) were used as control treatments. The sprayed plants were incubated at the temperature 24 °C and 12-h photoperiod. Injury level and biomass loss were observed 48 h after treatment. The rate of leaf damage was evaluated using visual scale from 0 (no symptoms) to 100% (all leaves are necrotic). Aerial shoots of the treated and control plants of *C. arvense* were cut and dried at room temperature to a constant weight. 

### 3.7. Data Analysis

All of the bioassays were performed three times. Data were subjected to one-way and two-way ANOVA followed by comparison of multiple treatment levels with the control applying the least significant difference (LSD) at *p* = 95%. All the statistical analyses were performed using Statistica 8.0 (StatSoft, Tusla, OK, USA).

## 4. Conclusions

Curvulin and phaeosphaeride A were isolated for the first time as phytotoxic metabolites from *Paraphoma* sp. VIZR 1.46 liquid and solid cultures, respectively. Curvulin showed a weak nonselective phytotoxic activity when assayed on ten plant species in a concentration of 8.4 × 10^−4^ M, matching data reported earlier for this compound [36,37,38,39]. Despite being nontoxic against the tested bacteria, fungi and paramecia, further research work with curvulin seems to be unpromising in light of the weak phytotoxicity of this compound. Phaeosphaeride A on the other hand demonstrated a high level of nonselective toxicity on all the tested plants. The phytotoxic activity of this compound identified as phyllostictine B was reported earlier when tested on Canada thistle at a concentration of 6 × 10^−3^ M [26]. It was shown to be inactive against the tested bacteria and fungi and weakly toxic against ciliates and these results are in accordance with data reported earlier [26]. Further studies are needed to elucidate the mode of action of phaeosphaeride A. Research is in progress to evaluate the possibility of enhancing the phytotoxic activity of this compound for intact plants as well to optimize its production. Our results have shown that addition of some adjuvants, including Hasten and Biopower, allowed increasing activity of the toxin on intact leaf discs of *C. arvense*. The herbicidal effect of the 0.5% semi-purified extract of solid culture of *Paraphoma* sp. VIZR 1.46 in combination with Hasten (0.1%) was strong causing fast leaf damage and 3-fold loss of dry mass of aerial shoots. These results can open the opportunity for further selection of appropriate adjuvants and their concentrations for increasing efficacy and potential of other natural herbicides.

## Supplementary Materials

Supplementary data are available online. Figure S1: A Bayesian 50% majority rule consensus tree based on dataset of LSU alignment of cf. *Paraphoma* sp. VIZR 1.46 and related species (1); Figure S2: A Bayesian 50% majority rule consensus tree based on dataset of LSU alignment of cf *Paraphoma* sp. VIZR 1.46 and related species (2); Figure S3: Point differences in nucleotide sequences of LSU for strains *Paraphoma* sp. 1.46, *P. chrysanthemicola* CBS 522.66, *P. radicina* CBS 102,875 and *P. vinacea* UMPV 003; Figure S4: UV spectrum of curvulin, dissolved in MeCN; Figure S5: ESI Mass spectrum of curvulin, recorded in positive mode; Figure S6: ^1^H-NMR spectrum of curvulin (CDCl_3_, at 400 MHz); Figure S7: ^13^C-NMR spectrum of curvulin (CDCl_3_, at 100 MHz); Figure S8: UV spectrum of phaeosphaeride A, dissolved in acetonitrile; Figure S9: ESI Mass spectrum of phaeosphaeride A , recorded in positive mode; Figure S10: ^1^H-NMR spectrum of phaeosphaeride A (DMSO *d*_6_, at 400 MHz); Figure S11: ^13^C-NMR spectrum of phaeosphaeride A (DMSO-*d*_6_, at 100 MHz); Figure S12: ^13^C-DEPT spectrum of phaeosphaeride A (DMSO-*d*_6_, at 100 MHz); Figure S13: ^1^H, ^1^H-COSY spectrum of phaeosphaeride A (DMSO *d*_6_, 100, 6 MHz); Figure S14: ^1^H, ^13^C-HMQC spectrum of phaeosphaeride A (DMSO *d*_6_); Figure S15: ^1^H, ^13^C-HMBC spectrum of phaeosphaeride A (DMSO-*d*_6_); Figure S16: ^1^H, ^1^H-ROESY spectrum of phaeosphaeride A (DMSO-*d*_6_, 400 MHz); Figure S17: (a) Structure of for phaeosphaeride A (2) (numbering of atoms is given according to to those given by Maloney et al., 2006 [41] Selected ROESY (^1^H-to ^1^H) NMR correlations; Table S1: ^1^H- and ^13^C-NMR data of phaeosphaeride A (2 in DMSO-*d*_6_); Table S2: Summary of three-way ANOVA analysis of the effect of adjuvants, solvents and wounding on phytotoxicity of phaeosphaeride A against *C. arvense*; Figure S18: Effect of adjuvants on herbicidal activity of 0.5% semi-purified extract (ca. 0.25% phaeosphaeride A) from solid state culture of *Paraphoma* sp. VIZR 1.46 on *Cirsium arvense* plants (48 h after treatment).

## Patents

The research resulted in two patents (RU No. 2596928 and RU No. 2619300).
