# Supplementary material for: Curvulin and Phaeosphaeride A from Paraphoma sp. VIZR 1.46 Isolated from Cirsium arvense as Potential Herbicides
Source: Molecules. 2018 Oct 28;23(11):2795. doi: 10.3390/molecules23112795 (PMC6278443; doi:10.3390/molecules23112795)
Supplement: Supplementary file 1 [file molecules-23-02795-s001.pdf]

Supporting Materials

Curvulin and Phaeosphaeride A from *Paraphoma* sp. VIZR 1.46 Isolated from *Cirsium arvense* as Potential Herbicides

Ekaterina Poluektova[a], Yuriy Tokarev[a], Sofia Sokornova[a], Leonid Chisty[b], Antonio Evidente[c], Alexander Berestetskiy[a]*

[a] All-Russian Institute of Plant Protection, Pushkin, Saint-Petersburg, 196608 Russian Federation
[b] Research Institute of Hygiene, Occupational Pathology and Human Ecology, Federal Medical Biological Agency, p/o Kuz'molovsky, Saint-Petersburg, 188663 Russian Federation
[c] Department of Chemical Sciences, University of Naples Federico II, Complesso Universitario Monte S. Angelo, Via Cintia 4, 80126 Napoli, Italy

LIST of Contents

Figure S1 - A Bayesian 50 % majority rule consensus tree based on dataset of LSU alignment. Bootstrap support values for maximum likelihood equal to or greater than 50 % are given above the nodes. The tree is rooted to *Stagonospora trichophoricola*
Figure S2 - A Bayesian 50 % majority rule consensus tree based on dataset of LSU alignment of cf *Paraphoma* sp. VIZR 1.46 and related species. Bootstrap support values for maximum likelihood equal to or greater than 40 % are given above the nodes. The tree is rooted to *Stagonospora trichophoricola*
Figure S3 - Point differences in nucleotide sequences of LSU for strains *Paraphoma* sp. 1.46, *P. chrysanthemicola* CBS 522.66, *P. radicina* CBS 102875 and *P. vinacea* UMPV 003

Figure S4 – UV spectrum of curvulin, dissolved in MeCN
Figure S5 – ESI Mass spectrum of curvulin, recorded in positive mode
Figure S6 - ^{1}H NMR spectrum of curvulin (CDCl$_3$, at 400 MHz)
Figure S7 – ^{13}C NMR spectrum of curvulin (CDCl$_3$, at 100 MHz)
Figure S8 - UV spectrum of phaeosphaeride A, dissolved in acetonitrile
Figure S9 - ESI Mass spectrum of phaeosphaeride A, recorded in positive mode
Figure S10 - ^{1}H -NMR spectrum of phaeosphaeride A (DMSO d-6, at 400MHz)
Figure S11 - ^{13}C -NMR spectrum of phaeosphaeride A (DMSO d-6, at 100 MHz)
Figure S12 - ^{13}C-DEPT spectrum of phaeosphaeride A (DMSO d-6, at 100 MHz)
Figure S13- ^{1}H,^{1}H COSY spectrum of phaeosphaeride A (DMSO d-6, 100,6 MHz)
Figure S14 - ^{1}H,^{13}C HMQC spectrum of phaeosphaeride A (DMSO d-6)
Figure S15 - ^{1}H,^{13}C HMBC spectrum of phaeosphaeride A (DMSO d-6)
Figure S16- ^{1}H,^{1}H ROESY spectrum of phaeosphaeride A (DMSO d-6, 400 MHz)
Figure S17 – a) Structure of for phaeosphaeride A (numbering of atoms is given according to to those given by Maloney et al. 2006 b) Selected ROESY (^{1}H to ^{1}H) NMR correlations
Table S1. -^{1}H and ^{13}C NMR data of phaeosphaeride A (in DMSOd-6)[a,b]
Table S2. Summary of three-way ANOVA analysis of the effect of adjuvants, solvents and wounding on phytotoxicity of phaeosphaeride A against *Cirsium arvense*
Figure S 18 – Effect of adjuvants on herbicidal activity of 0.5% semi-purified extract (ca. 0.25% phaeosphaeride A) from solid state culture of *Paraphoma* sp. VIZR 1.46 on *Cirsium arvense* plants (48 hours after treatment)

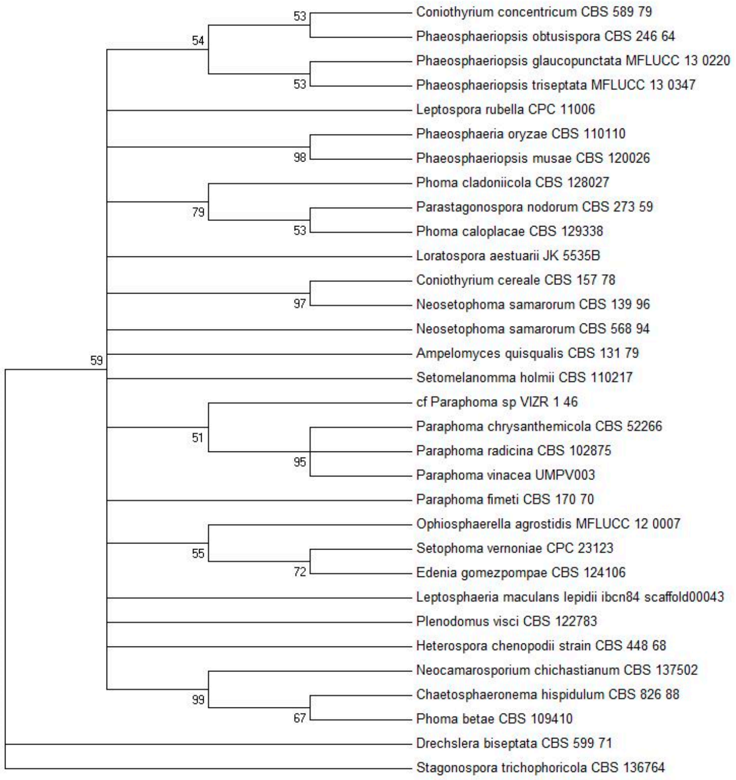

Figure S1 - A Bayesian 50 % majority rule consensus tree based on dataset of LSU alignment. Bootstrap support values for maximum likelihood equal to or greater than 50 % are given above the nodes. The tree is rooted to *Stagonospora trichophoricola*

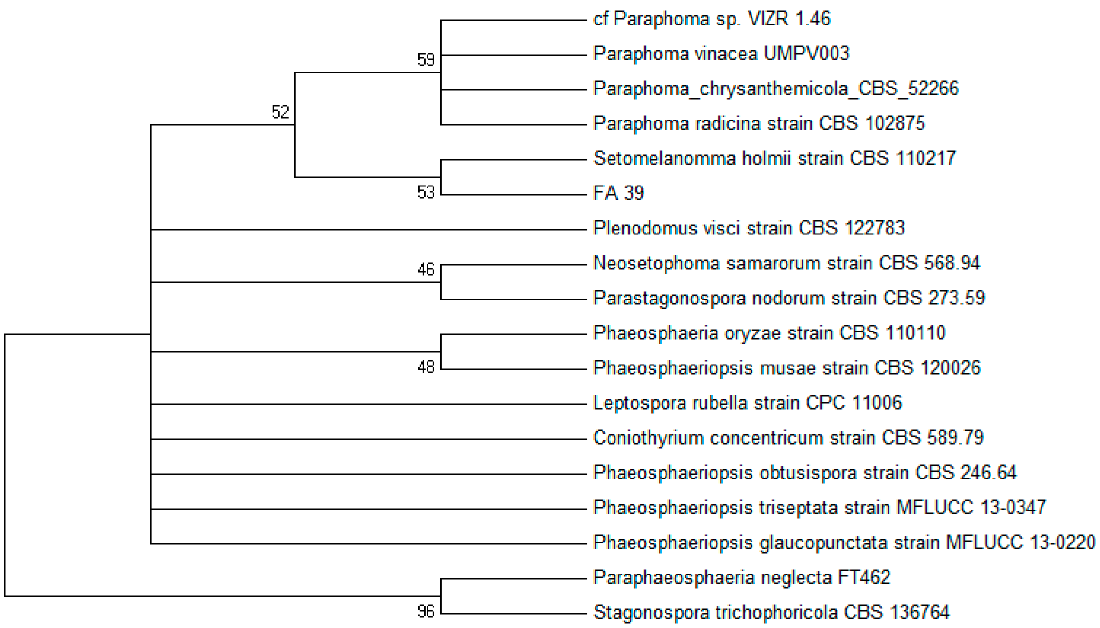

Figure S2 - A Bayesian 50 % majority rule consensus tree based on dataset of LSU alignment of cf *Paraphoma* sp. VIZR 1.46 and related species. Bootstrap support values for maximum likelihood equal to or greater than 40 % are given above the nodes. The tree is rooted to *Stagonospora trichophoricola*

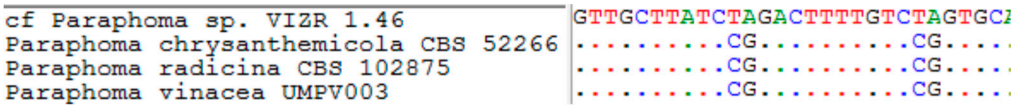

Figure S3 - Point differences in nucleotide sequences of LSU for strains *Paraphoma* sp. 1.46, *P. chrysanthemicola* CBS 522.66, *P. radicina* CBS 102875 and *P. vinacea* UMPV 003

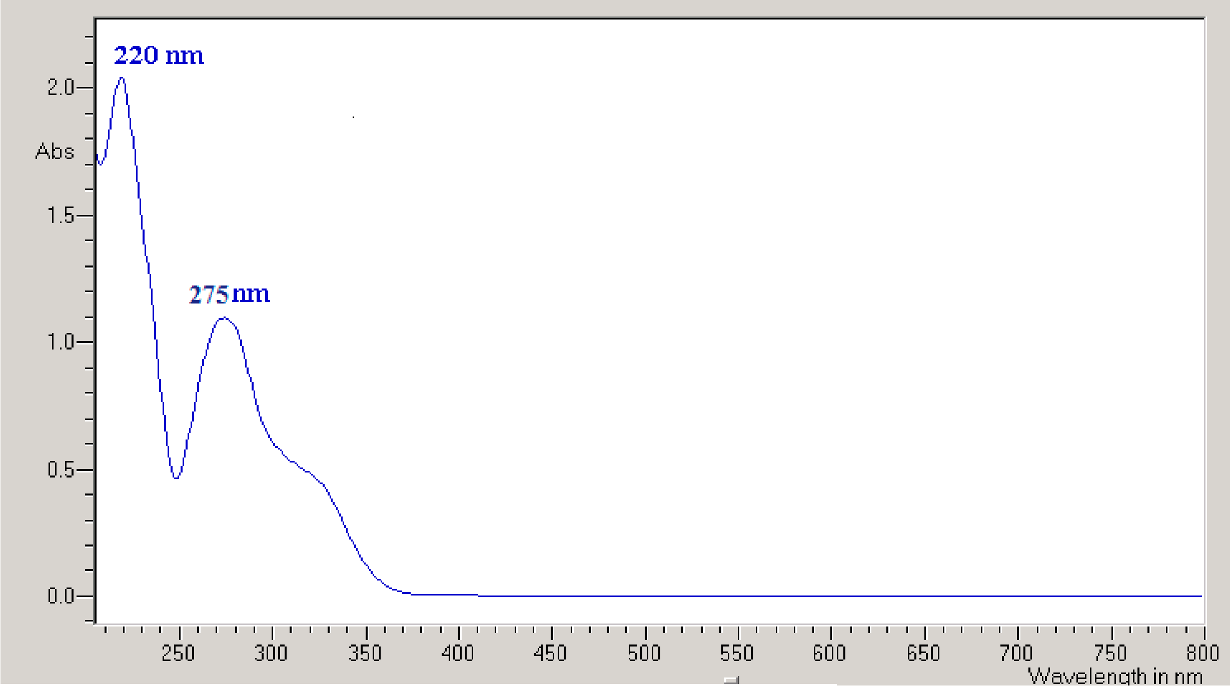

Figure S4 – UV spectrum of curvulin, recorded in MeCN

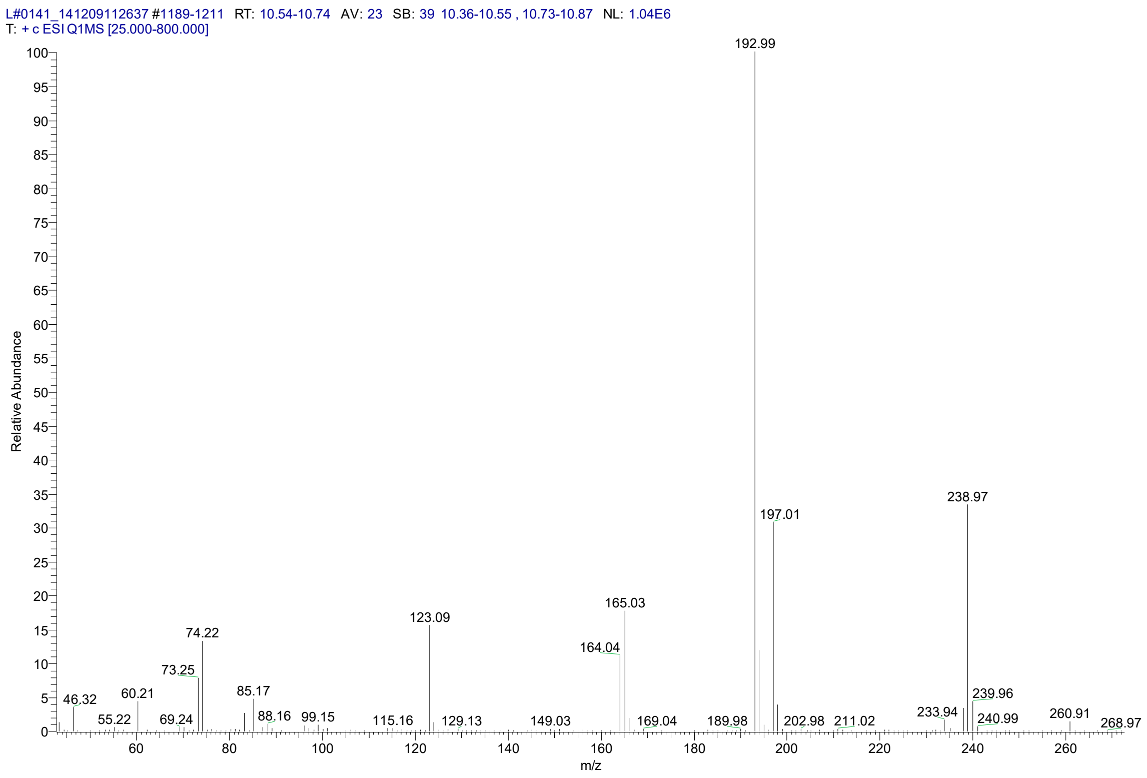

Figure S5 – ESI Mass spectrum of curvulin, recorded in positive mode

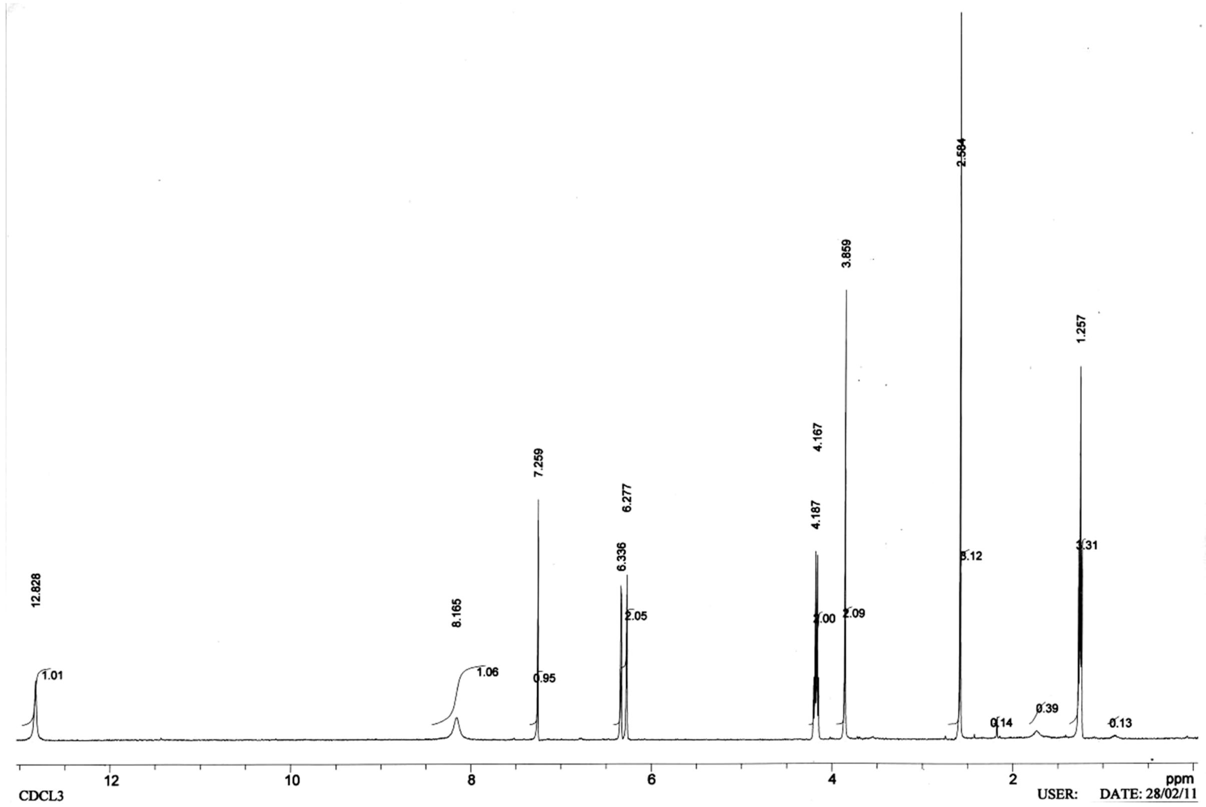

Figure S6 - ^{1}H-NMR spectrum of curvulin ($CDCl_3$, 400 MHz)

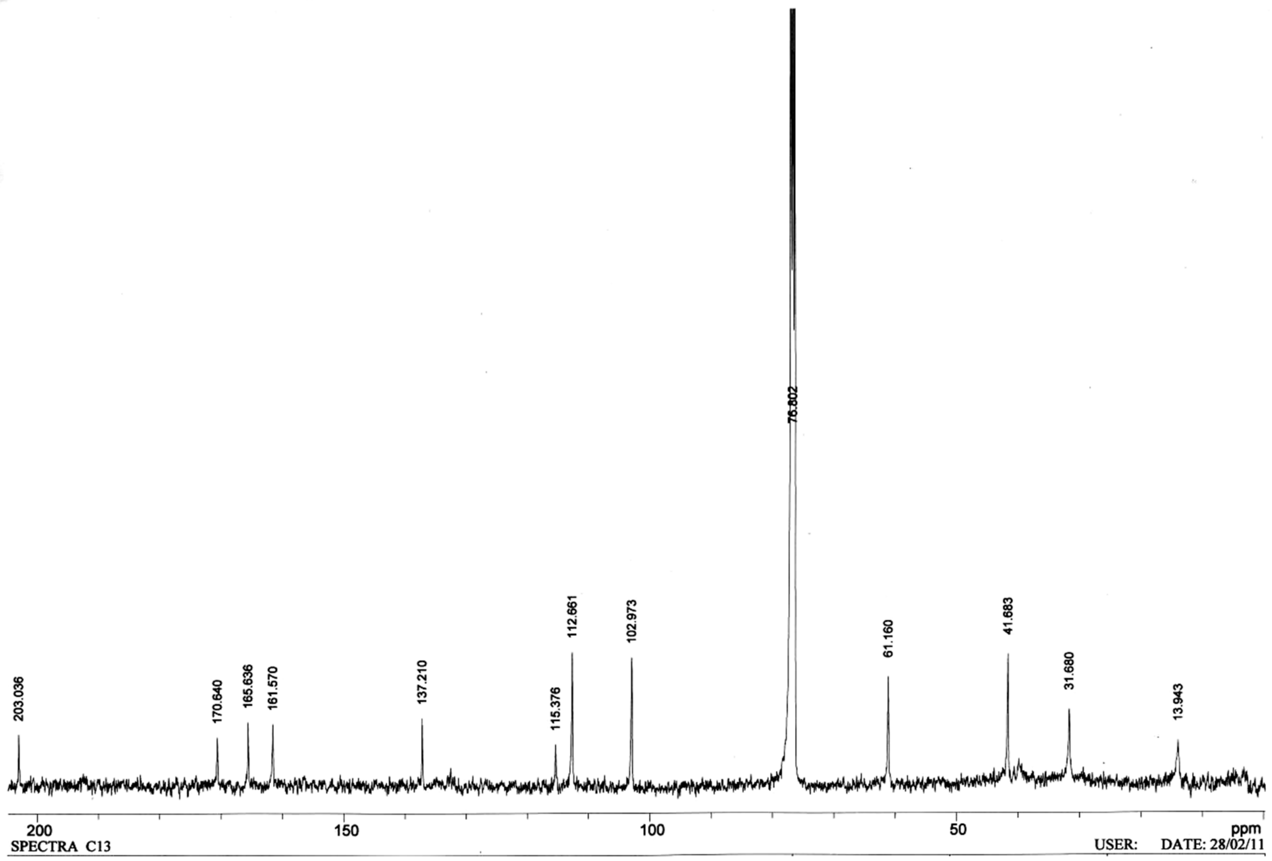

Figure S7 – ^{13}C-NMR spectrum of curvulin ($CDCl_3$, 100 MHz)

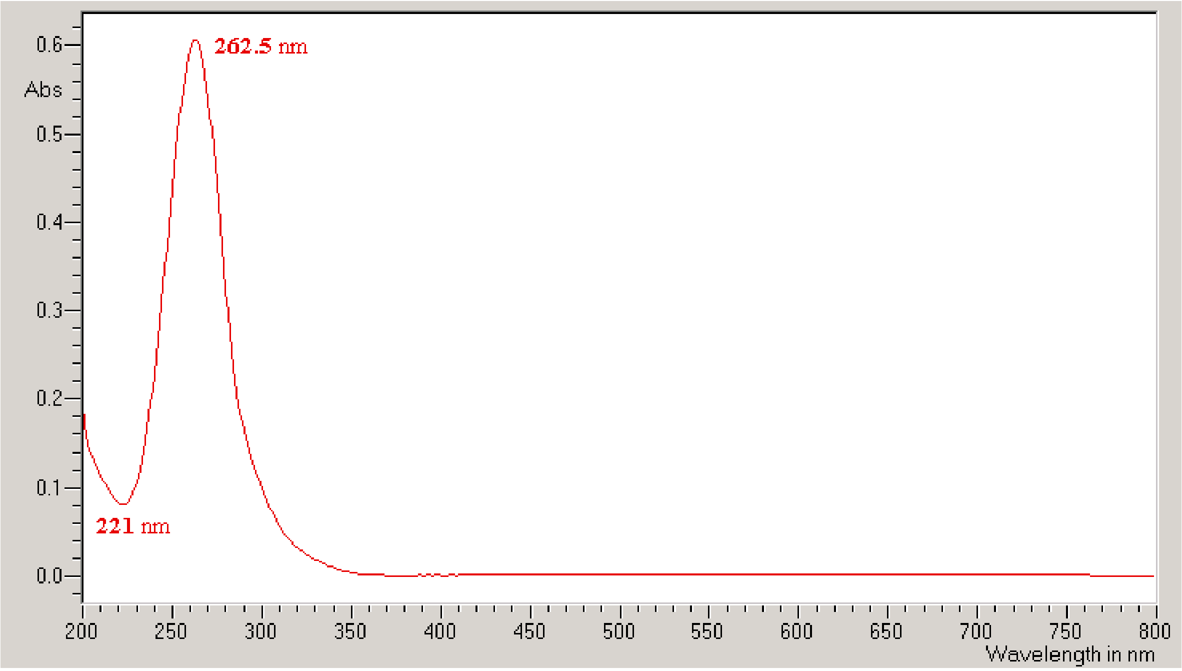

Figure S8 – UV spectrum of phaeosphaeride A, dissolved in MeCN

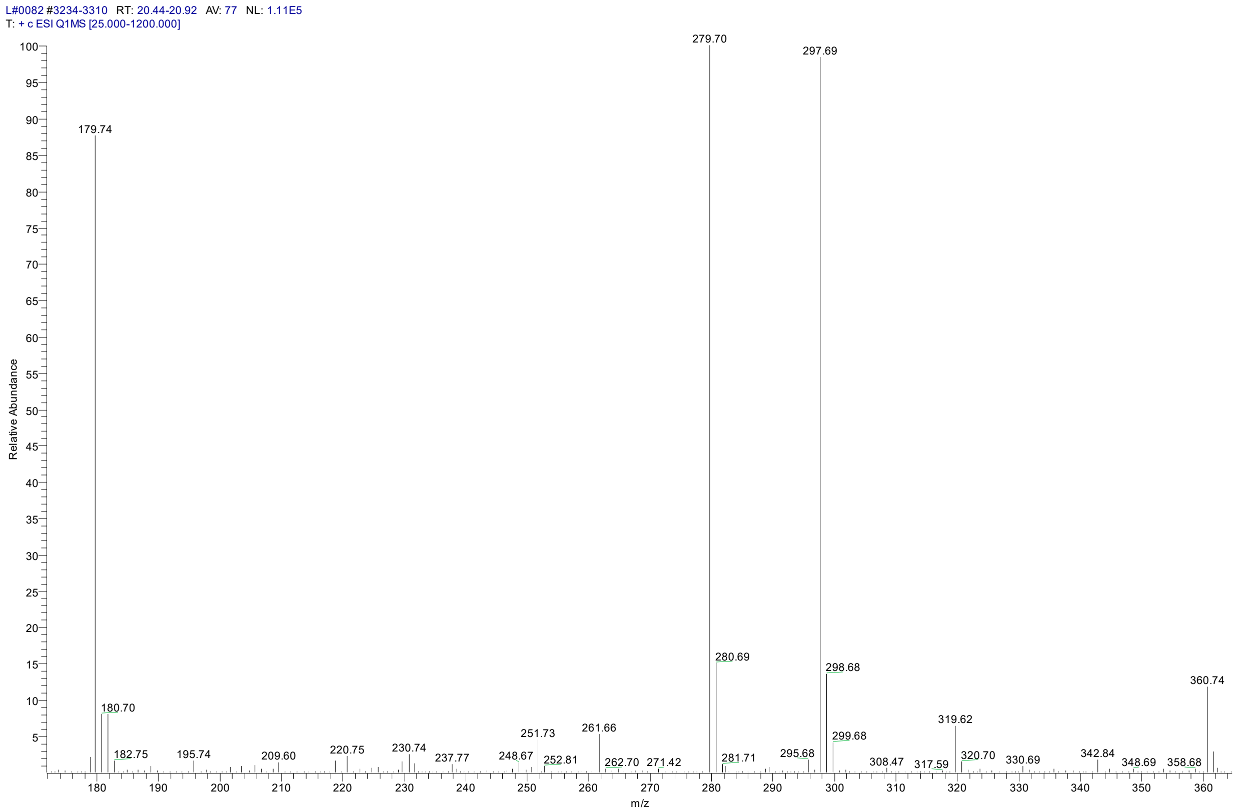

Figure S9 – ESI Mass spectrum of phaeosphaeride A, recorded in positive mode

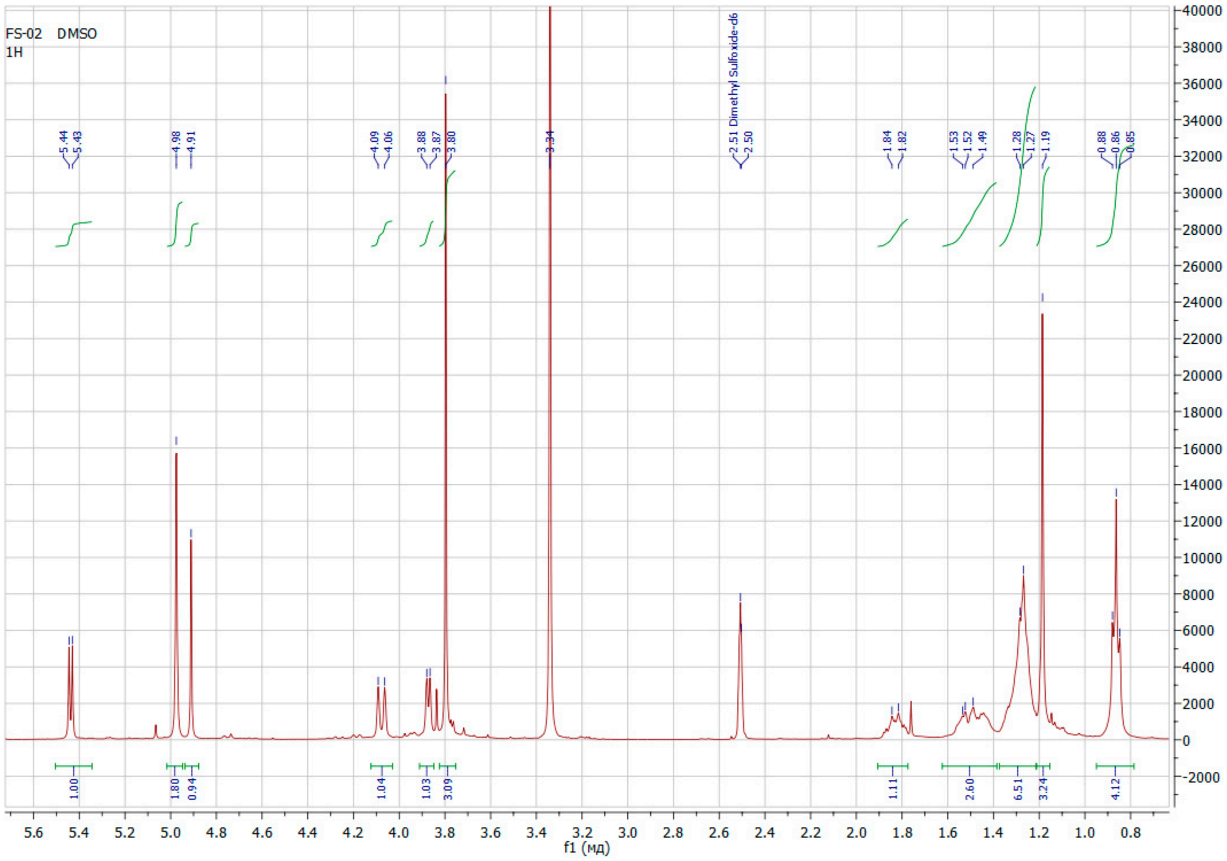

Figure S10 - ^{1}H-NMR spectrum of phaeosphaeride A (DMSO d-6, at 400 MHz)

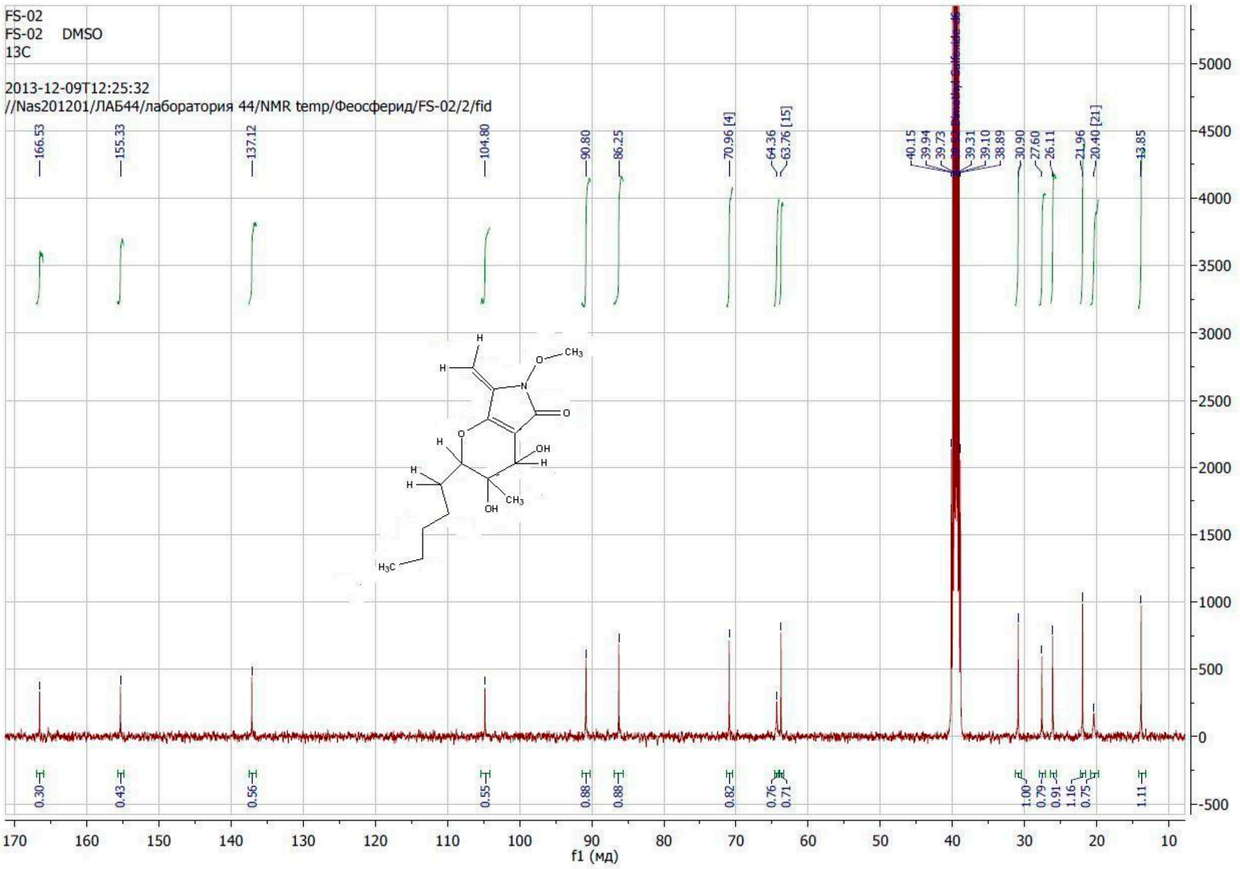

Figure S11 - ^{13}C -NMR spectrum of phaeosphaeride A (DMSO d-6, at 100 MHz)

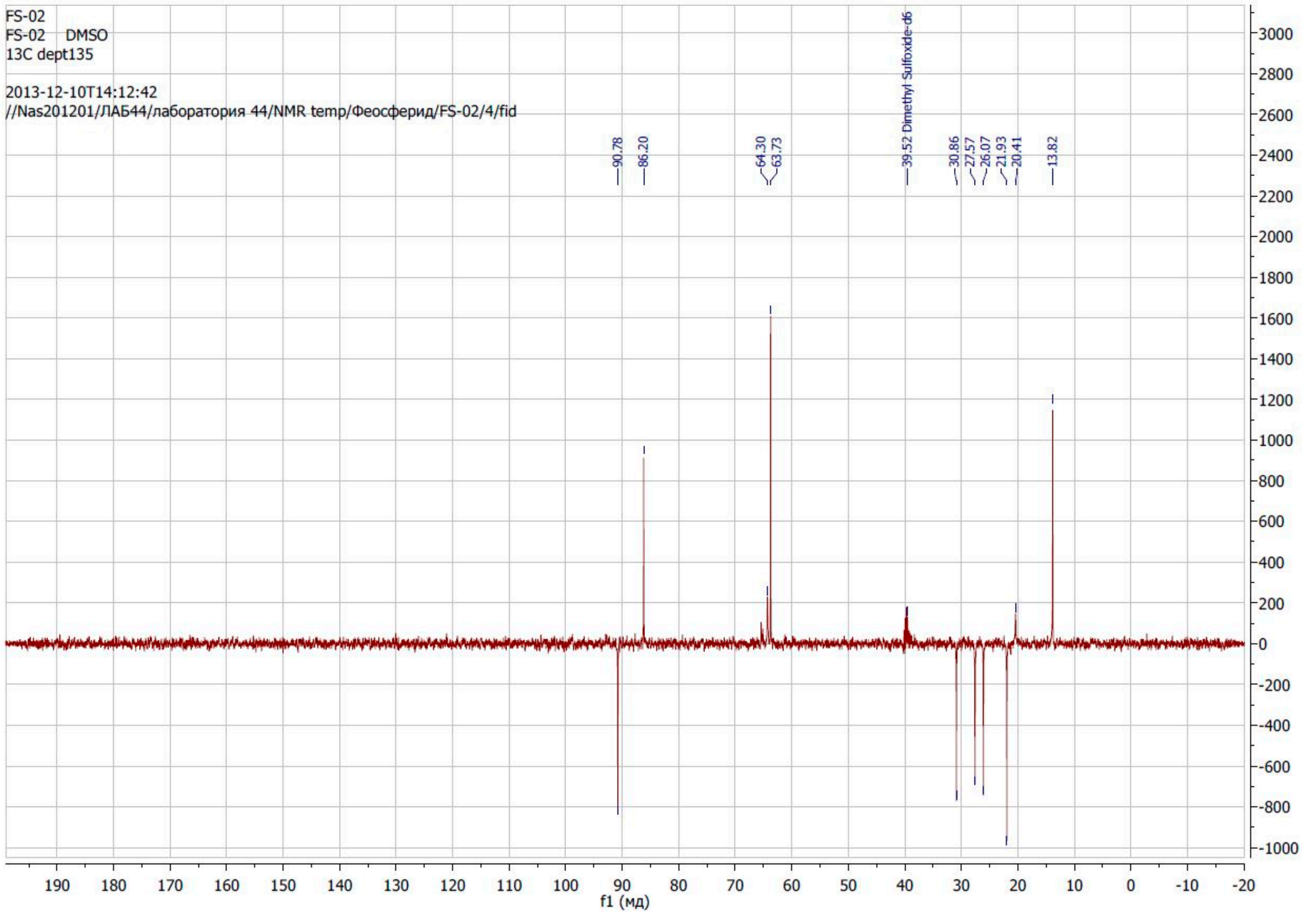

Figure S12 - ^{13}C DEPT spectrum of phaeosphaeride A (DMSO d-6, at 100 MHz)

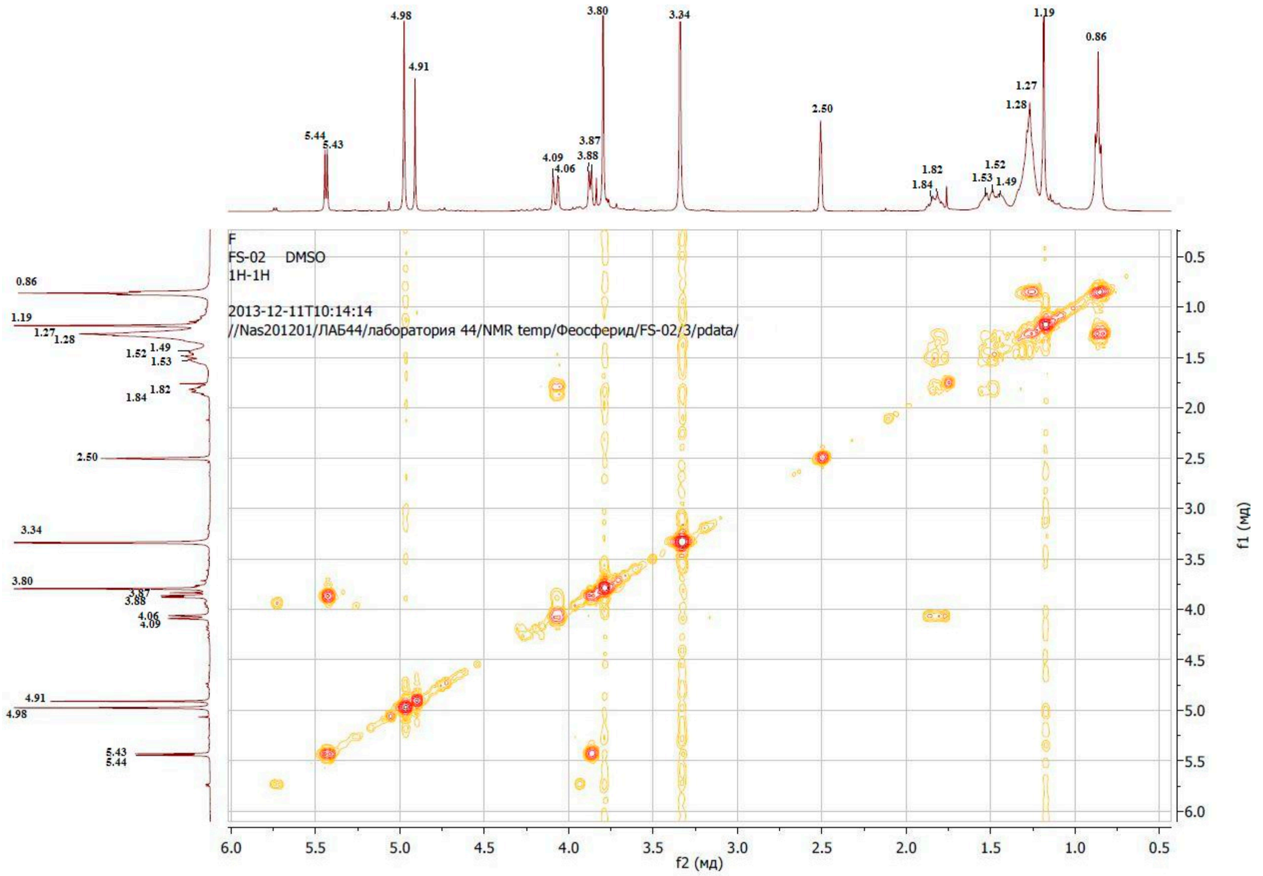

Figure S13 - ^{1}H,^{1}H COSY spectrum of phaeosphaeride A (DMSO d-6, at 100 MHz)

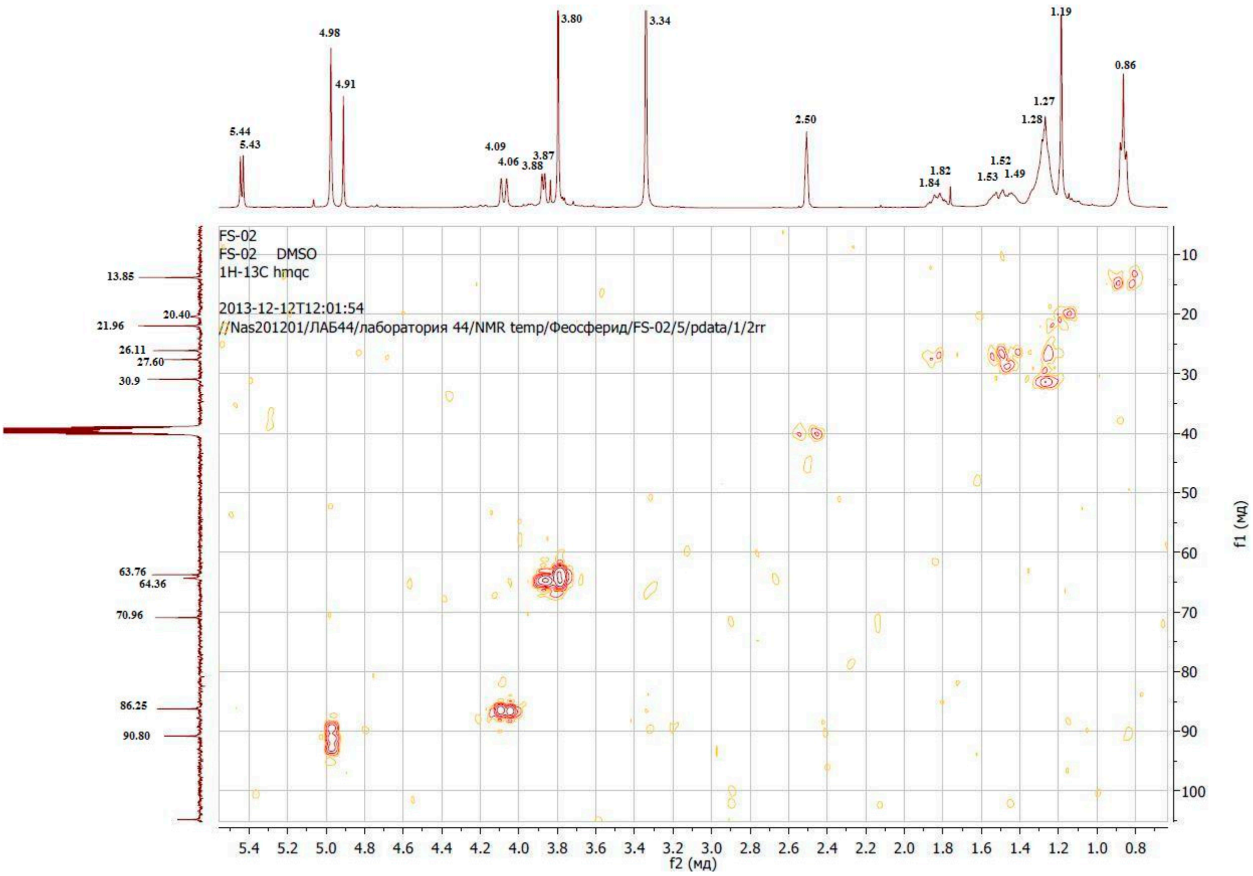

Figure S14 - $^{1}H,^{13}C$ HMQC spectrum of phaeosphaeride A (DMSO d-6)

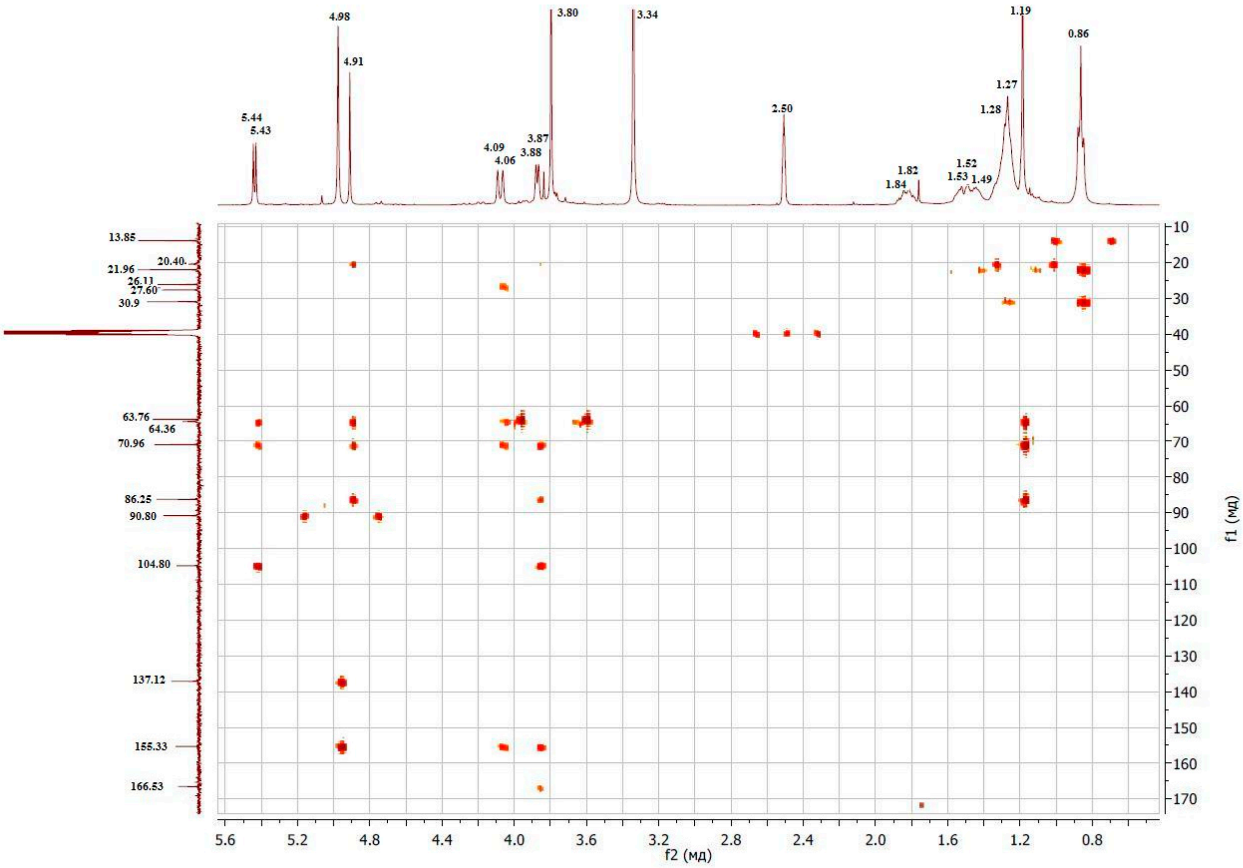

Figure S15 - ^{1}H,^{13}C HMBC spectrum of phaeosphaeride A (DMSO d-6)

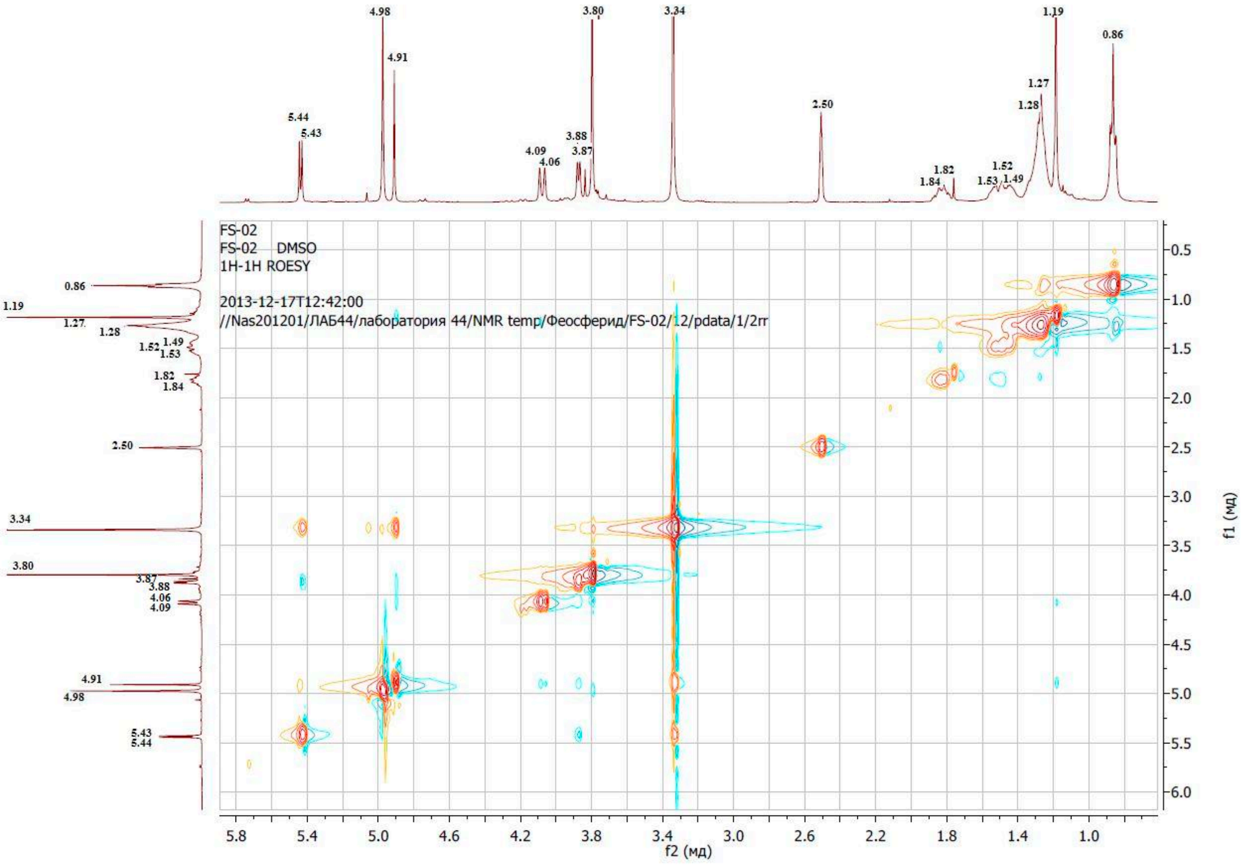

Figure S16 - $^{1}H,^{1}H$ ROESY spectrum of phaeosphaeride A (DMSO d-6, 400 MHz)

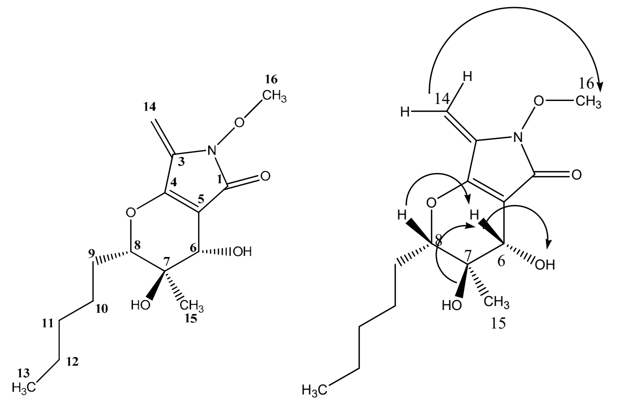

a b

Figure S 17 - a) Structure of phaeosphaeride A (**2**) (numbering of atoms is given according to those given by Maloney et al. 2006; b) Selected ROESY (^{1}H to ^{1}H) NMR correlations

Table S1. -^{1}H and ^{13}C NMR data of phaeosphaeride A (in DMSOd-6)[a,b]

Position	δC[c]	δH (J in Hz)	HMBC
1	166.5 s	-	
3	137.1 s	-	H_2-14
4	155.3 s	-	H-6, H_2-14
5	104.8 s	-	H-6
6	64.4 d	3.87 (d, 4.0)	
7	71.0 s	-	HO-6
8	86.3 d	4.08 (d, 12 Hz,)	HO-6, HO-7, H-6, H_2-11
9	27.6 t	1.82 (m)1.52 (m)	
10	26.1 t	1.49 (m, 2H)	
11	30.9 t	1.27 (m, 2H)	
12	22.0 t	1.28 (m, 2H)	
13	13.9 q	0.86 (t, 6.4 Hz, 3H)	
14	90.8 t	4.98 (s, 2H)	
15	20.4 q	1.19 (s, 3H)	HO-7
16	63.8 q	3.80 (s, 3H)	
HO-6		5.42 (d, 4.0)	
HO-7		4.92 (s, 1H)	

[a]The chemical shifts are in δ values (ppm) from TMS. [b]2D ^{1}H,1H (COSY) ^{13}C,^{1}H (HMQC) NMR experiments delineated the correlations of all the protons and the corresponding carbons. [c]Multiplicities were assigned by DEPT spectrum.

Table S2. – Summary of three-way ANOVA analysis of the effect of adjuvants, solvents and wounding on phytotoxicity of phaeosphaeride A against *Cirsium arvense*

Factors	SS	DF	MS	F	p
adjuvant	17,0727	5	3,4145	7,509	0,000001
solvent	1,4592	1	1,4592	3,209	0,074387
wounding	132,7092	1	132,7092	291,833	0,000000
adjuvant x solvent	8,6860	5	1,7372	3,820	0,002335
adjuvant x wounding	3,5335	5	0,7067	1,554	0,173474
solvent x wounding	8,9253	1	8,9253	19,627	0,000014
adjuvant x solvent x wounding	4,7507	5	0,9501	2,089	0,067078
Error	120,0525	264	0,4547		

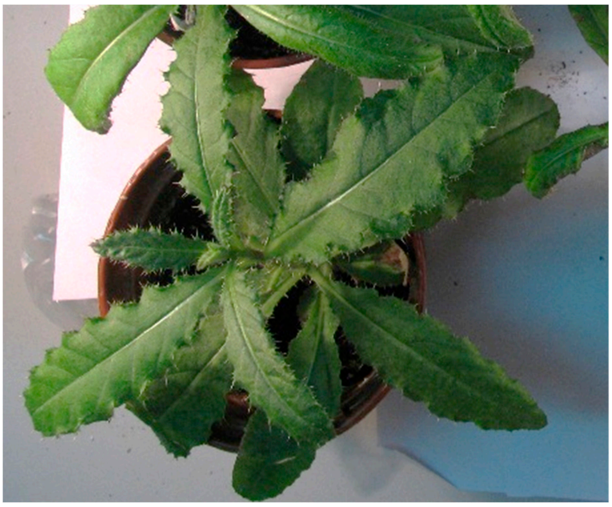

Control

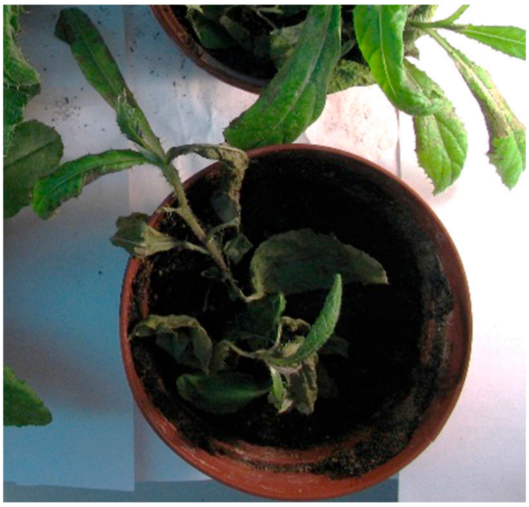

Phaeosphaeride A (ca. 0.25%) + Hasten (0.1%)

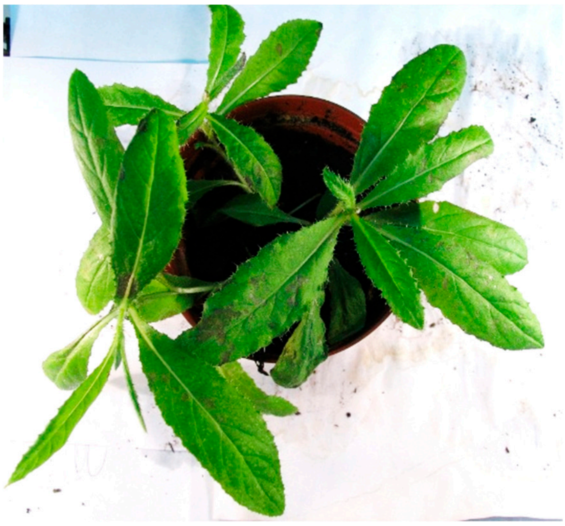

Phaeosphaeride A (ca. 0.25%) + water

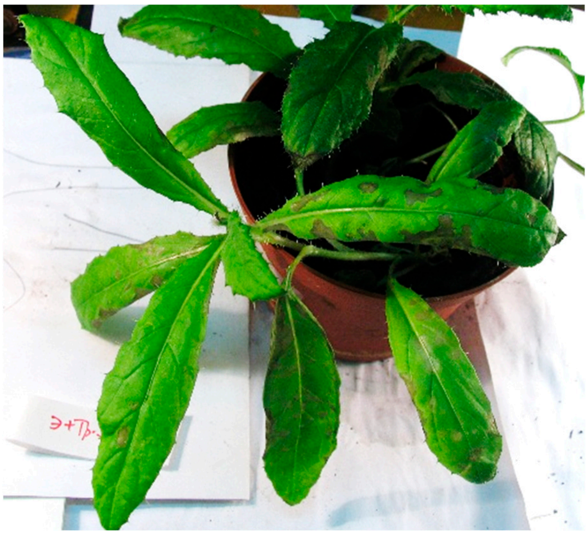

Phaeosphaeride A (ca. 0.25%) + Biopower (0.1%)

Figure S 18 – Effect of adjuvants on herbicidal activity of 0.5% semi-purified extract (ca. 0.25% phaeosphaeride A) from solid state culture of *Paraphoma* sp. VIZR 1.46 on *Cirsium arvense* plants (48 hours after treatment)
